# A new species of *Macrocypraea* (Gastropoda, Cypraeidae) from Trindade Island, Brazil, including phenotypic differentiation from remaining congeneric species

**DOI:** 10.1371/journal.pone.0225963

**Published:** 2020-01-08

**Authors:** Luiz Ricardo L. Simone, Daniel C. Cavallari

**Affiliations:** 1 Museu de Zoologia da Universidade de São Paulo, São Paulo, Brazil; 2 Faculdade de Filosofia, Ciências e Letras de Ribeirão Preto da Universidade de São Paulo, Ribeirão Preto, SP, Brazil; University of California, UNITED STATES

## Abstract

*Macrocypraea mammoth* is a new species from Trindade, a remote oceanic island located 1160 km off Espírito Santo, Brazil. This isolated species is described in a detailed morphological scenario that includes all Recent congeneric species. The detailed anatomy of two Recent species, *M*. *zebra* and *M*. *cervinetta*, were described in a previous paper. The remaining one, *M*. *cervus*, is included herein. Brief taxonomical comments on all these species is also included. The new species can be distinguished from other Western Atlantic species by its larger size, proportionally heavier and more solid shell, more rounded and wider outline, longer posterior tapered ending and slightly inflated base; anatomically it has some exclusivities, such as the mantle papillae mostly bearing 3–5 distal aligned projections, osphradium with a shorter branch, modifications in some odontophore muscles, penis with a clear glandular region, and a sac-like bursa copulatrix with a long duct. Based on the comparative analysis, the genus *Macrocypraea* can be defined by the wide distance between the osphradium and gill; a twofold buccal muscle (mc); a radular ventral tensor muscle (pair m11) surrounding radular sac; and a bursa copulatrix located at middle level of the pallial oviduct. Register ZooBank: urn:lsid:zoobank.org:pub:C8E6E515-508F-47DE-9753-4EA619C1DDFD.

## Introduction

**Fig 1:**
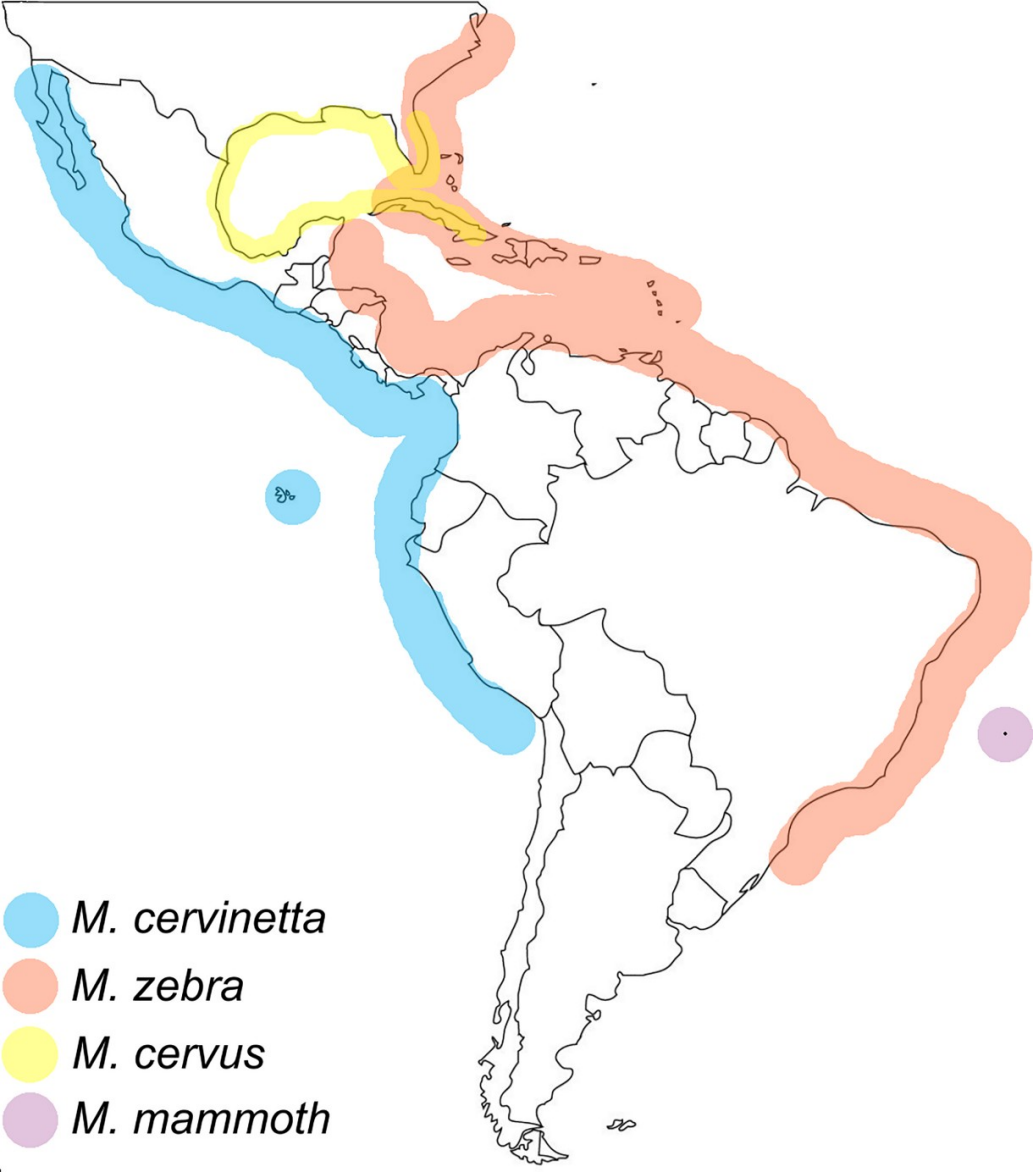
Schematic map of Americas showing approximate extension of the four studied species, color as indicated.

The cypraeid genus *Macrocypraea* was introduced by Schilder [1] in a footnote as a replacement name for *Erythraea* Mörch, 1877, which was preoccupied by Sowerby (1839). It currently encompasses three extant species, namely *Macrocypraea zebra* (Linné, 1758), *M*. *cervus* (Linné, 1771), and *M*. *cervinetta* (Kiener, 1844) (Fig 1). Recent studies have recognized several subspecies within the group: two subspecies for *M*. *cervus*, including the nominal subspecies ranging from the Upper Caribbean to the Gulf of Mexico, and *M*. *c*. *lindseyi* Petuch, 2013 restricted to southern Cuba; two for *M*. *zebra*, including the nominal subspecies ranging from Florida to the Caribbean, and *M*. *z*. *dissimilis* (Schilder, 1924) distributed along the Brazilian coastline; and two for *M*. *cervinetta*, from the Pacific, namely *M*. *c*. *cervinetta* ranging from Mexico to the western coast of South America, and *M*. *c*. *californica* Lorenz, 2017 from the Gulf of California. The genus also currently encompasses two subgenera, including the nominal subgenus, and the monotypic *Lorenzicypraea* Petuch & Drolshagen, 2011 with *M*. *cervus* as its type species. The genus only occurs in the Tropical and Subtropical coasts of the Americas.

The shell of *Macrocypraea* is mainly characterized by being dorsoventrally compressed with fine, darker-colored apertural teeth, well-developed fossula and columellar sulcus, dark brown dorsum, brown color in general (usually darker around the spire) with dispersed pale spots dorsally and laterally [2, 3]. Anatomically, a set of eight synapomorphies was reported by Simone [3, 4] as exclusive of *Macrocypraea* based only on *M*. *zebra* and *M*. *cervinetta*, as follows (characters and states from [3], in parenthesis): mantle papillae simple, broad, base narrow (30/4); osphradium separated from anterior region of gill (44/1); presence of two bundles of buccal muscles “mc” (67/1); odontophore pair of ventral tensor muscles of the radula m11 with insertion surrounding ventral region of radular sac base (70/2); odontophore pair m7 originating at anterior border of ventral m4 branch (80/1); dorsal inner folds of buccal mass with oblique secondary furrows in their anterior region (94/2); bursa copulatrix U-shaped, bearing inner folds (120/1), located in middle of pallial oviduct (121/1).

Trindade is an oceanic island located ca. 1160 km off the coast of Espírito Santo, southeastern Brazil (20°30'30″S 29°19′30″W) (Fig 1). Together with Martim Vaz and three lesser islands, Trindade is an emerged portion of the Vitória-Trindade Chain, a ~6 million-year-old series of extinct underwater volcanoes and guyots spread along the Brazilian shelf. These islands are known to harbor endemic marine mollusk species, e.g., *Leucozonia ponderosa* Vermeij & Snyder, 1998, and *Lottia marcusi* (Righi, 1966), among others [5, 6, 7]. Since its discovery in the early 16^th^ century, Trindade has received scientific expeditions from many different countries [8]. Material collected in some of these expeditions (e.g., Marion Dufresne MD55) has been described in recent papers and revealed several new mollusk species (e.g., [9]). Four of the latest expeditions carried out by different Brazilian research teams from 2011 to 2015 recovered many live specimens, including three individuals and several shells of a particularly large new species of Macrocypraea, which is introduced herein. Samples of the new species, even shell fragments, are very rare. Finding live specimens is an extraordinary event, which was only made possible after an intense survey during the last expedition.

In a previous phylogeny of the Cypraeoidea, Simone [3] described the anatomy of *M*. *zebra* and *M*. *cervinetta*. The present paper supplements that study with brief taxonomical comments and by thoroughly describing the anatomy of two additional species, including the new species from Trindade Island and *M*. *cervus*, which was not anatomically described before. These descriptions provide a more accurate scenario for the introduction of the new species, which is addressed herein, with remarks on the genus level. This paper only deals with fossil *Macrocypraea* species for comparative purposes. The subspecific level is not also addressed here, which would demand a different set of samples and approaches that are out of our scope.

## Material and methods

Specimens studied herein are adult and subadult individuals recovered by four recent expeditions to Trindade (2011–2015), with the addition of the *Macrocypraea* specimens previously studied by Simone [3]. Additional specimens from older expeditions (1916–1976) and private and museum collections were also examined. Recent specimens are deposited in the Museu de Zoologia da Universidade de São Paulo (MZSP); older items came from the Museu Nacional da Universidade Federal do Rio de Janeiro (MNRJ). Samples of similar species analyzed here are also from the MZSP collection and come from different localities along the Western Atlantic. Abbreviations used for shell measurements: H, height; L, length; W, width.

Specimens used in the CT-Scan analysis were scanned at the Centro para Documentação da Biodiversidade, University of São Paulo, Ribeirão Preto, Brazil, using a GE Phoenix v|tome|x s 240 Industrial High-Resolution CT & X-Ray System. High-resolution x-ray computed tomographies were obtained through a 240 kV high-power microfocus source, using the following settings: no filter, digital high-contrast detector DXR250RT, source operating at 80 Kv and 200 mA, 1 x 1 binning, source to object distance (FOD) 426.149 mm, source to detector distance (FDD) 812.4319 mm, 5 frames averaged, 1 skip frames, 1,300 projections, 333.09 ms timing, default offset correction, default gain correction. Resulting images were 32-bit grayscale and 990 (width) by 1000 pixels (height). Three-dimensional reconstruction was performed with GE Phoenix Datos X2. Visualization, editing, and segmentation of reconstructions of the CT models were carried out in VGStudio Max® V3.0.

Abbreviations in the figures: **aa**, anterior aorta; **ag**, albumen gland; **an**, anus; **ap**, aperture of pallial gonoducts; **ar**, anterior projection of head-foot; **as**, anal siphon; **au**, auricle; **bc**, bursa copulatrix; **ce**, cerebral ganglion; **cg**, capsule gland; **cm**, columellar muscle; **cv**, ctenidial vein; **dd**, duct to digestive gland; **df**, dorsal fold of buccal mass; **dg**, digestive gland; **di**, diaphragmatic septum; **ec**, esophageal gland; **ef**, esophageal folds; **eg**, secondary esophageal gland; **es**, esophagus; **ey**, eye; **ff**, furrowed part of dorsal folds of buccal mass; **fs**, foot sole; **ft**, foot; **gi** gill; **gp**, parietal ganglion; **hg**, hypobranchial gland; **in**, intestine; **ki**, kidney; **km**, membrane between kidney and pallial cavity; **kv**, ventral lobe of kidney; **ll**, left mantle lobe; **m1** to **m14**, extrinsic and intrinsic odontophore muscles; **mb**, mantle border; **mj**, jaws, buccal, and oral tube muscles; **mo**, mouth; **mp**, mantle papilla; **ne**, nephrostome; **ng**, nephridial gland; **nr**, nerve ring; **ns**, nephridial gland vessel inserted at right from nephrostome; **nv**, nerve; **oc**, odontophore cartilage; **od**, odontophore; **os**, osphradium; **ot**, oral tube; **oy**, ovary; **pb**, proboscis; **pc**, pericardium; **pd**, penis groove; **pe**, penis; **pg**, pedal glands anterior furrow; **pl**, penis gland; **pm**, pallial muscle (derived from columellar muscle); **pn**, pedal ganglion; **ps**, pallial spermgroove; **ra**, radula; **rh**, rhynchostome; **rl**, right mantle lobe; **rm**, retractor muscle of proboscis; **rn**, radular nucleus; **rr**, anterior triangular sinus of visceral mass connected to pallial floor; **rs**, radular sac; **rt**, rectum; **sa**, salivary aperture; **sc**, subradular cartilage; **sd**, salivary duct; **sg**, salivary gland; **si**, siphon; **st**, stomach; **su**, subesophageal ganglion; **sv**, seminal vesicle; **te** cephalic tentacle; **tg**, integument; **ts**, testis; **vd**, vas deferens; **vi**, visceral ganglion; **vo**, visceral oviduct.

### Systematics

#### Genus *Macrocypraea* Schilder, 1930

*Macrocypraea* Schilder, 1930: 55. Lorenz, 2071[2]: 286.

Type species: *Cypraea exanthema* Linné, 1767 (= *M*. *zebra* (L., 1758) see [10, 11]) by original designation. Recent, Western Atlantic.

#### *Macrocypraea mammoth* sp. nov.

**Fig 2:**
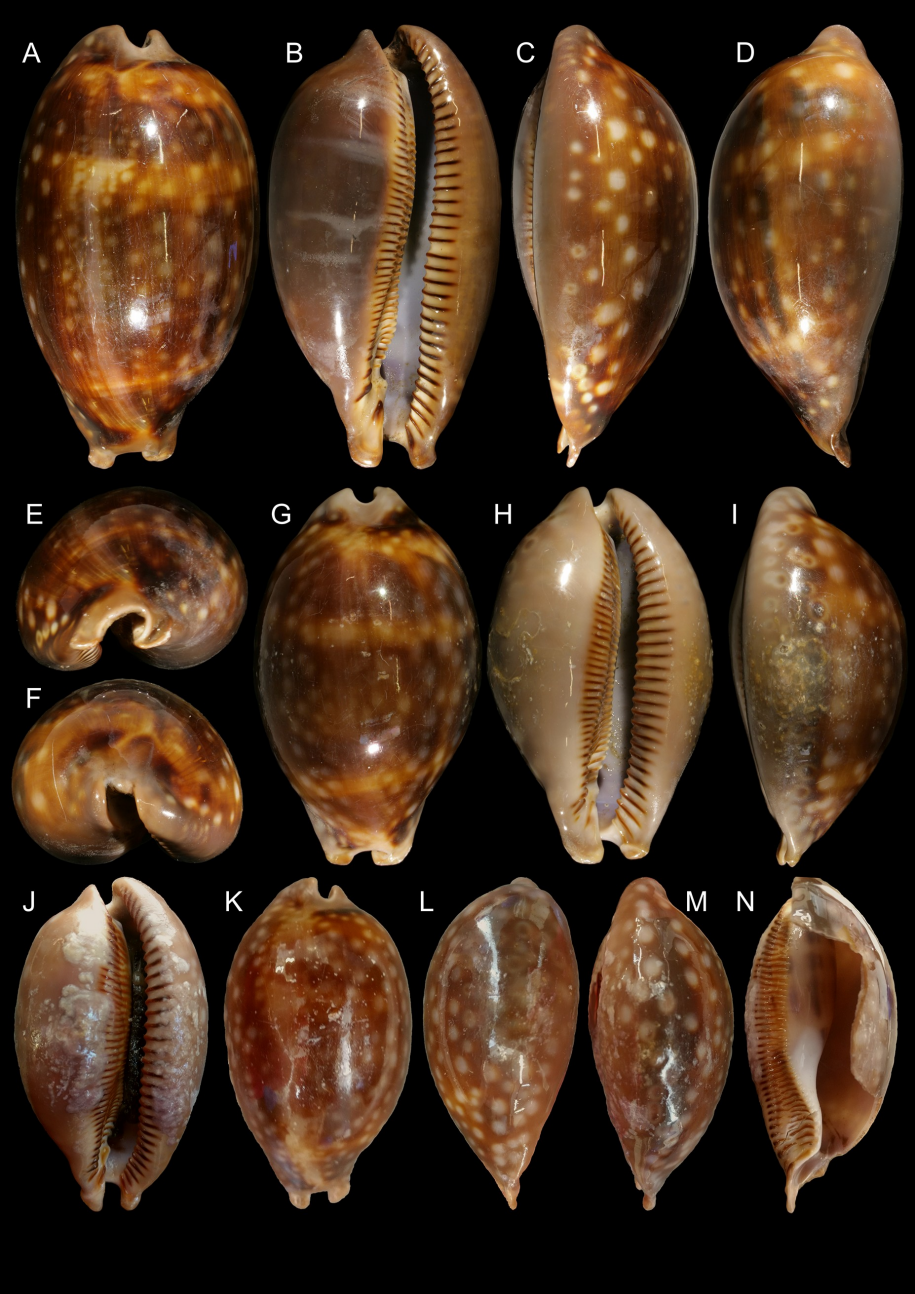
*Macrocypraea mammoth* shells of types. (A-F) Holotype MZSP 137544 (L 133.1 mm). (A) Dorsal view. (B) Apertural view. (C) Right view. (D) Left view. (E) Anterior view. (F) Posterior view. (G-I) paratype MZSP 137545 (L 100.0 mm). (G) Dorsal view. (H) Apertural view. (I) right view. (J-N) Paratype MZSP 137546 (now broken) (L 112.3 mm). (J) Apertural view. (K) Dorsal view. (L) Left view. (M) Right view. (N) Same, right portion of shell removed.

**Fig 3:**
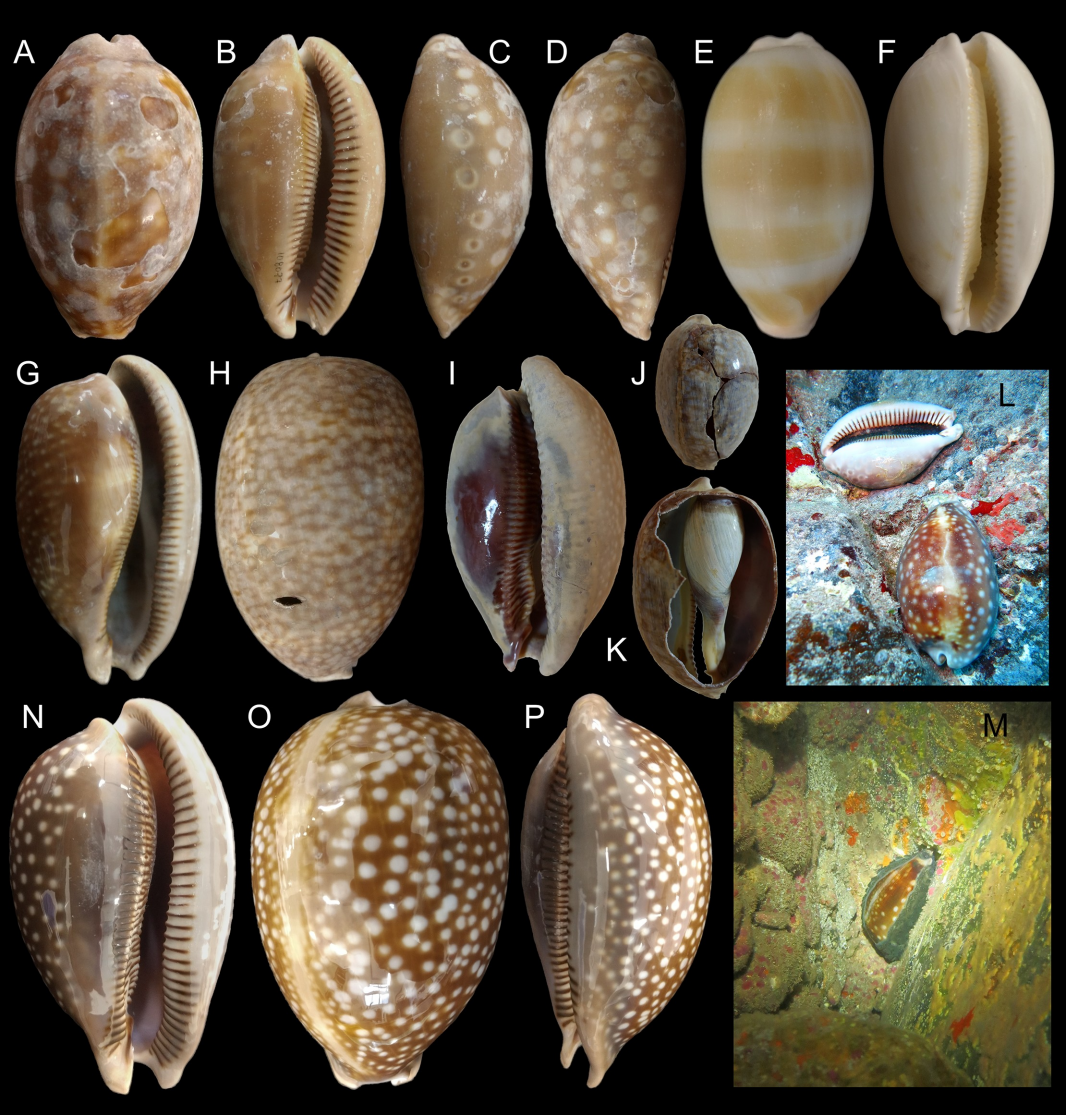
*Macrocypraea* spp shells and in situ. (A-D) *M*. *mammoth* paratype MZSP 108077 (L 95.8 mm). (A) Dorsal view. (B) Apertural view. (C) Right view. (D) Left view. E) *M*. *mammoth* MZSP 108079 subadult specimen (L 80.4 mm), dorsal view. (F) Same, apertural view. (G) *M*. *cervus* MZSP 8149, from Mexico (L 152.9 mm), Apertural view. (H) Same, dorsal view. (I-K) *M*. *cervus* ANSP 93276, from Florida (L 129.4 mm). (I) Apertural view. (J) Dorsal view (broken). (K) Same, specimen and dorsal shell wall removed. (L) Alive paratypes in situ.; (M) Alive holotype in situ. (N-P) *M*. *cervus*, MZSP 115362, from Cuba (L 98.5 mm). (N) Apertural view. (O) Dorsal view. (P) Right view.

**Fig 4:**
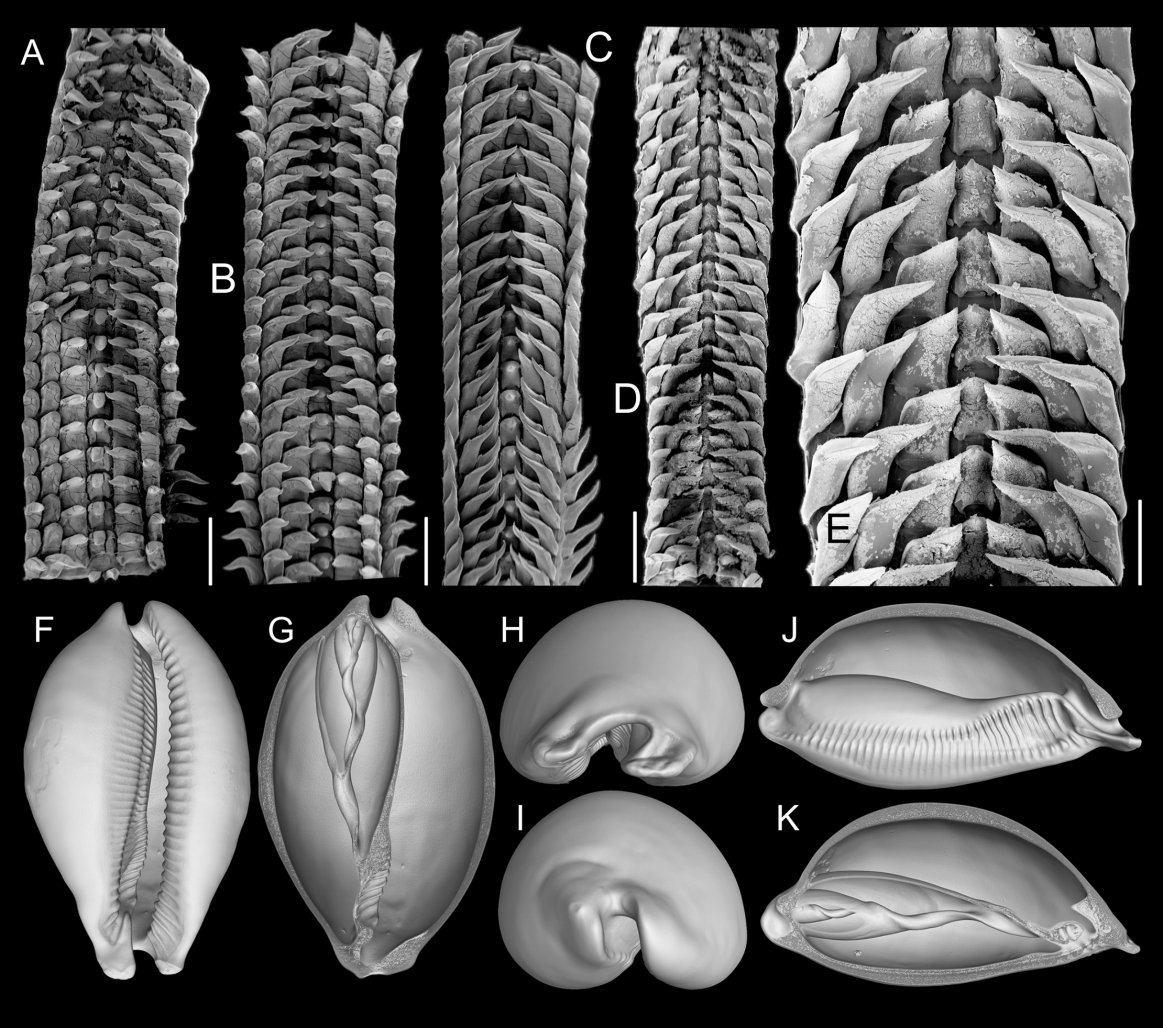
*Macrocypraea* spp radulae in SEM. (A) *M*. *mammoth* paratype MZSP 137546; (B-C) *M*. *mammoth* holotype. (D) *M*. *cervus* ANSP 93276. (E) Same, detail. scales = 32–35: 1 mm, 36: 0.5 mm. (F-K) *M*. *mammoth* MZSP 137545, 3D reconstructions of shell (CT-Scan) (L 100.0 mm). |(F) Frontal view. (G) Same, more ventral portions removed. (H) Anterior view. (I) Posterior view. (J) Right view, right half removed. (K) Same, section along columellar axis.

**Fig 5:**
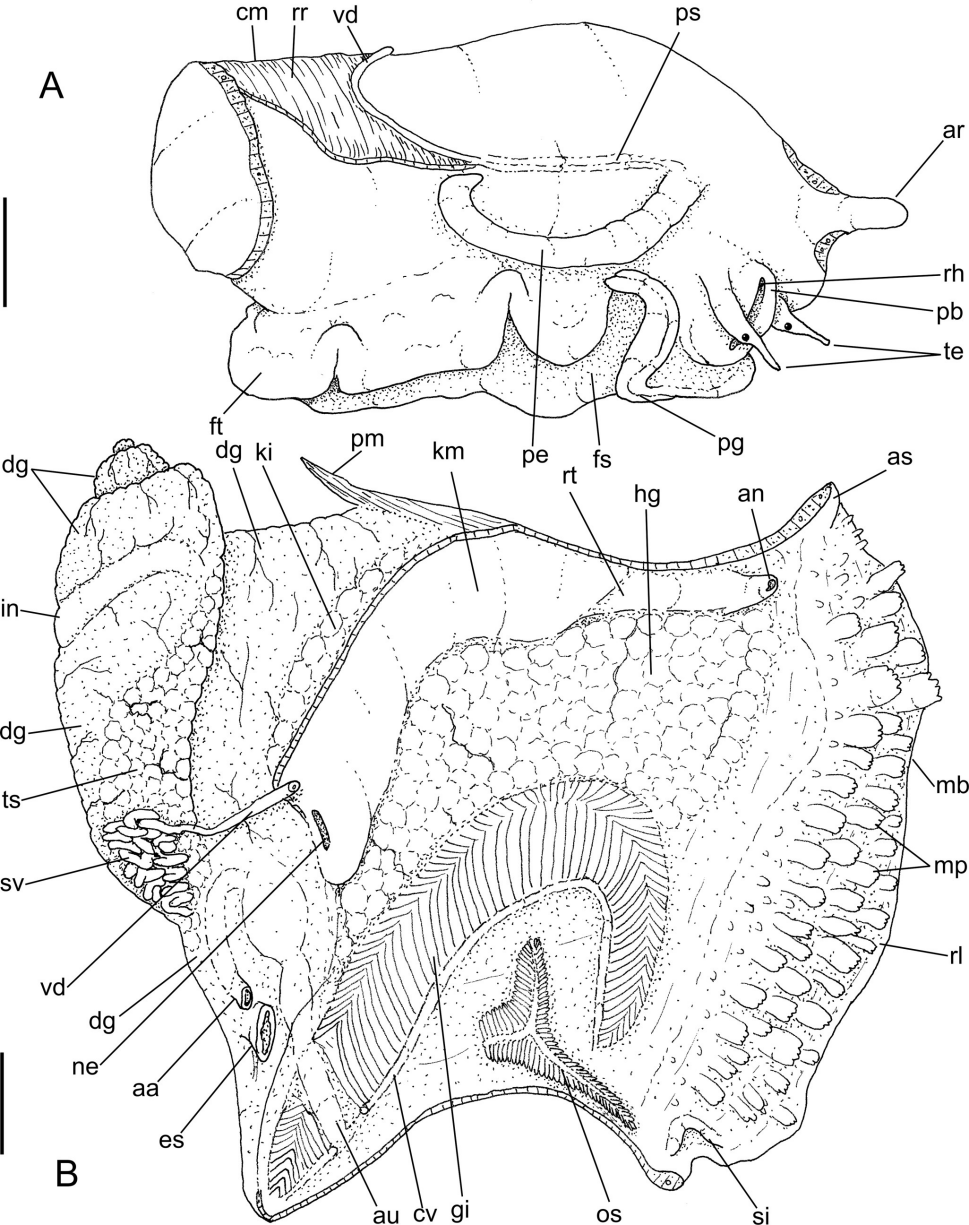
*Macrocypraea mammoth* anatomy. (A) Head-foot, male, right view. (B) Pallial roof and visceral mass, ventral view, portion of gill ventral to pericardium removed. Scales = 10 mm.

**Fig 6:**
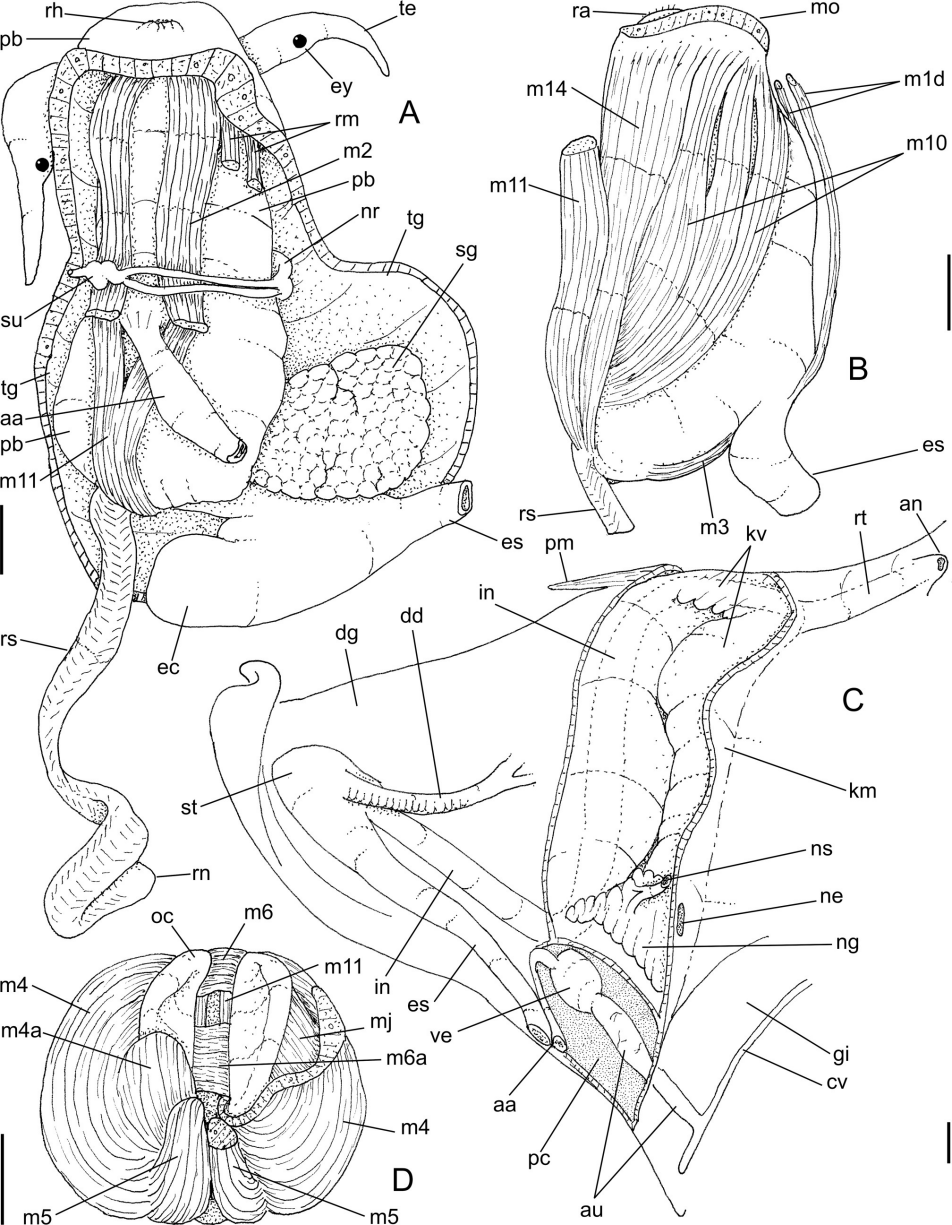
*Macrocypraea mammoth* anatomy. (A) Head, ventral view, foot and columellar muscle removed, radular sac uncoiled downwards. (B) Buccal mass, left view. (C) Visceral mass and posterior part of pallial cavity, ventral view, midgut seen as in situ, kidney opened longitudinally, only topology of gill shown. (D) Odontophore, dorsal view, both cartilages (oc) deflected, left m4 and m5 (right in figure) partially sectioned. Scales = 5 mm.

**Fig 7:**
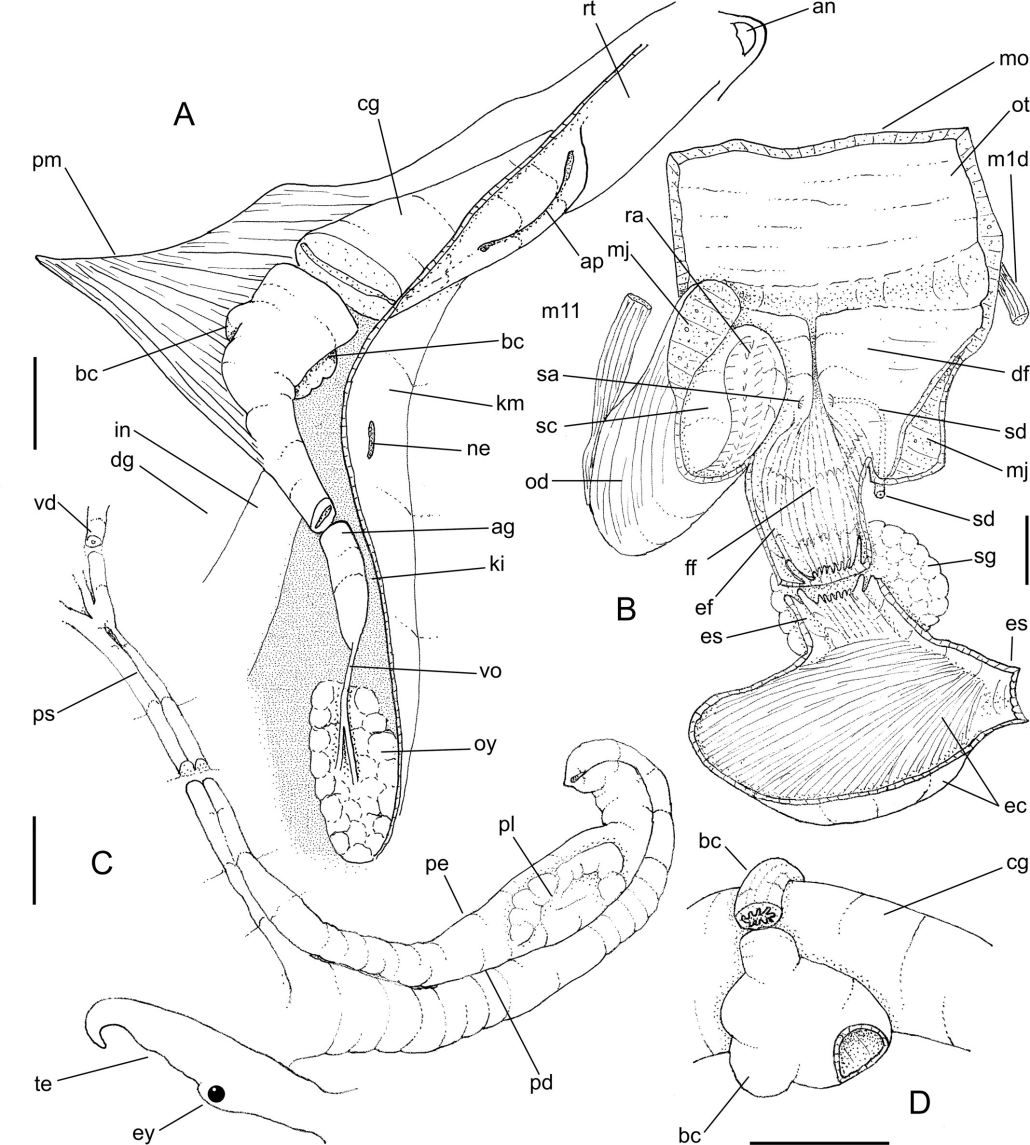
*Macrocypraea mammoth* anatomy. (A) Female genital system and adjacent structures as in situ, ventral view, transverse section artificially done in middle region of pallial oviduct, ovary (oy) only partially shown, kidney not shown in details. (B) Foregut, ventral view, opened longitudinally, odontophore (od) deflected to left. (C) Anterior male genital structures, dorsal view, topology of right tentacle (te) also shown. (D) Detail of middle region of pallial oviduct, dorsal view, bursa copulatrix (bc) artificially sectioned in two regions. Scales = 5 mm.

(Figs 2 and 3F, 3L–3M and 4–7)

urn:lsid:zoobank.org:act:B76C5BC6-595A-4782-A706-6A2BBF1D718F

*Cypraea zebra*: Rios, 1987[12]: 59 (partim) (non Linnaeus, 1758).

*Cypraea (Macrocypraea) zebra*: Leal, 1991[13]: 99; Rios, 2009[14]: 123, text figure. (partim) (non Linnaeus, 1758).

*Macrocypraea zebra dissimilis*: Lorenz, 2017[2]: 289, text figure., pl. 59 (partim) (non Schilder, 1924).

#### Type specimens

Holotype ♀, MZSP 137544. Paratypes: BRAZIL: off **Espírito Santo**; Trindade Island: Praia dos Cabritos, MNRJ 5059, 1 shell (B. Prazeres col., xii.1975-ii.1976), Calhaus/Farrilhões, MNRJ 5056, 1 shell (J. Becker & A.B. Costa col., 1965, sta. D248/966), Calheta, MZSP 108077, 20°30’37.6”S 29°18’28.1”W, 1 shell (J.B. Mendonça col., 12.vi.2012), MZSP 137545, 20°30’29.5”S 29°18’37.0”W, 16.3 m, 1 ♂, MZSP 137546, 1 ♂ (shell now broken) (J.B. Mendonça col., 9.vii.2015).

#### Type locality

BRAZIL, off Espírito Santo state, Trindade Island, Secon/GCIT, 20°30’20.9”S 29°18’43.7”W, 11.6 m (J.B. Mendonça col., 9.vii.2015).

#### Diagnosis

Shell large, solid and heavy. Posterior ending tapered and long, base slightly inflated. Anterior end flattened, projected, flanking both sides of siphon. Color pattern with large, scattered beige spots, most basal of which presenting dark brown central area. Teeth of inner lip slope long, close from each other, radially disposed, forming nodes preceding canal. Mantle papillae mostly bearing 3–5 small aligned distal projections. Osphradium elongated, with short posterior branch and narrow anterior branch. Odontophore protractor muscles (m1d) composed of single strong dorsal pair of bundles; ventral protractor pair (m10) with multiple branches. Seminal vesicle with uniform width along its length; pallial sperm groove with similar-sized edges. Penis with clear glandular region. Bursa copulatrix bearing long duct, sac-like, with thin-walled terminal region.

### Description

#### Shell (Figs 2–4)

Large (95–133 mm), inflated, antero-posteriorly elongated, solid and heavy, with tapered anterior and posterior extremities, 1.8 times as long as wide, slightly taller (~10%) than wide (Figs 2A, 2G, 2K, 3A and 4H). Surface glossy, color light to dark brown, with lighter-colored longitudinal median dorsal line (usually dislocated to right), and scattered rounded beige spots; spots near base usually larger, some with dark brown (ocellated) central area (Fig 2C, 2I and 2M). Basal area usually lighter colored, especially around aperture, with dark brown labral teeth (Figs 2B, 2I, 2J and 3B); younger individuals ground color yellowish, with wide orange transverse stripes dorsally (Fig 3E and 3F). Aperture slit-like, long and narrow, becoming progressively wider toward anterior end (Figs 2B, 2H, 2J and 4F); width ~12–15% of shell width. Inner lip glossy, bearing 35–42 strong labral teeth, each individual tooth sometimes branching into 2–3 narrower teeth towards interior of aperture; inner slope full of narrow, transverse teeth, radially disposed (virtual focus above middle region of dorsal shell surface; Fig 4J); anterior plate relatively deep concave, tall (~half local height), ventral end of more anterior teeth strongly nodose, with a node at base of ventral-left siphonal edge (Fig 4J). Outer lip bearing 34–41 strong labral teeth. Canal flanked by pair of spatulate long projections (Figs 2E and 4G), length ~1/6 of remaining shell length (Fig 4F) relatively horizontal positioned (Fig 4J and 4K). Shell walls relatively thick (Fig 4G, 4J and 4K).

Shell variation relatively small. Size normally over 130 mm, but some rare specimens are about 90 mm. pale spots usually small, uniformly splayed (Fig 2A, 2G and 2K), but sometimes spots larger (Fig 3A, 3C and 3D). Ocellated spots usually visible in right side (Figs 2C, 2I, 2M and 3C), rather than left side (Fig 2D and 2L), with exceptions (Fig 3D).

#### Head-foot (Figs 5A and 6A)

Exposed areas homogeneous dark grayish-brown. Proboscis cylindrical, large, occupying ~70% of haemocoel volume (Fig 6A). Cephalic tentacles long, origin located approximately at ventral lateral surface of snout base (Fig 5A: **te**); proximal half distinctly broader than distal half. Anterior furrow of pedal gland (**pg**) well-developed. Columellar muscle (cm) short (~half whorl long), thin distally. Anterior projection of head solid-muscular (Fig 5A: **ar**), originating in nuchal region, bending toward anterior region, located at left of siphonal canal. Accessory muscle of columellar muscle running along right edge of main muscle, attached to pallial cavity, right margin gradually fading (Fig 5B: **pm**). Haemocoel relatively broad, elliptical. Haemocoel connection with visceral mass (esophageal and aortic passage) located at middle level of left margin of haemocoel (Fig 6A). Two pairs of small retractor muscles of proboscis (Fig 6A: **rm**) originating in ventro-lateral middle region of haemocoel, bordering lateral haemocoel inner surface towards anterior region, inserting along ventral and lateral wall of proboscis; median pair larger than remaining counterparts.

#### Mantle organs (Fig 5B)

Mantle border very large, broad, with left and right lobes (**rl**) exceeding shell aperture, covering shell completely during activity. Outer surface of exposed part of mantle dark grayish-brown in color (Fig 3M), bearing several uniformly distributed papillae (**mp**), except for anterior and posterior regions. Each papilla with base narrower than its middle region, profile broad, rather cylindrical, narrowing distally, with 3–5 transversally-aligned small terminal projections. Siphon small (**si**) distinctly separated from mantle border, shallow, lacking marginal papillae. Anal siphon (**as**) also separated from mantle border, slightly shallower and locaded on opposite side of incurrent siphon. Osphradium (**os**) occupying ~1/15 of pallial cavity roof, relatively large, located at some distance from siphon. Osphradium with 3 branches; anterior branch turned forward toward siphon, length ~70% of osphradium length, narrow, pointed; right branch similar to anterior branch, but slightly broader and shorter; left branch with ~1/3 of anterior branch length, distal end blunt, rounded. Each osphradium branch bipectinate, each filament low and thin, its base almost fully connected to mantle, except for rounded tip bent externally. Osphradium ganglion thick, running along central region of each branch. Distance between osphradium and gill equivalent to osphradium width. Gill (**gi**) occupying ~1/3 of pallial cavity roof, long, strongly curved (concavity located to left), surrounding osphradium. Ctenidial vein relatively broad, width homogeneous along its length; connection with auricle sub-terminal (Fig 5B: **au**) (described below). Each gill filament tall, triangular, tip long, slender and sharp (Fig 5B). Distance between gill and visceral mass narrow in posterior half, successively far from visceral mass and rectum in anterior half. Hypobranchial gland very developed (**hg**), thick, white; occupying area between gill and rectum in anterior 2/3 of pallial roof. Hypobranchial gland supported by transverse, thin septa originating from adjacent mantle. Rectum relatively broad, running along right margin of cavity. Anus siphoned, located close to anal siphon. Genital gonoducts running ventral and at right from rectum, most in floor of pallial cavity (details below).

#### Visceral mass (Figs 5B and 6C)

Relatively small, ~3 whorls posterior to pallial cavity. Right anterior region more anterior than left one for ~1/2 whorl, invading pallial cavity. Gonad (**ts**) occupying from second whorl up to pericardial region, surrounding digestive gland on columellar side. Stomach with adjacent esophagus and intestine obliquely occupying central region of first visceral whorl. Digestive gland (**dg**) dark brown, filling space between stomach (and adjacent digestive tubes) and gonad, being more massive anteriorly to stomach in last visceral whorl. Kidney (**ki**) and pericardium as anterior border of digestive gland.

#### Circulatory and excretory systems (Fig 6C)

Heart relatively large, located in anterior-left region of visceral mass (Fig 5B). Auricle elongated, narrow, connecting with ctenidial vein at about ~1/10 before its posterior end, running immersed in mantle, crossing obliquely and dorsally to gill (Fig 5B: **au**), running about equivalent distance inside pericardial cavity, becoming slightly broader toward its insertion in ventricle. Ventricle large (**ve**), walls thick muscular. Aortas running attached to left pericardial wall, anterior aorta (**aa**) ~3 times broader than posterior aorta, running parallel to esophagus. Anterior aorta within haemocoel easily visible up to buccal mass (Fig 6A: **aa**). Kidney narrow, long, located in central and right regions of posterior limit of pallial cavity (Fig 5B). Kidney chamber mostly hollow, intestine running along its dorsal inner surface, digestive gland located in its posterior limit. Kidney lobe (**kv**) single, as solid glandular mass in right-anterior region of kidney, attached to adjacent intestine; gradually becoming flattened mass attached to membrane between kidney and pallial chambers. Nephridial gland triangular (**ng**), occupying ~1/4 of kidney inner space, transversely folded; central vessel well-developed (**ns**), running on right side, ventral to nephrostome. Nephrostome (**ne**) as broad, transverse slit in left region of membrane between kidney and pallial cavity (**km**), close to pericardium.

#### Digestive system (Figs 6B–6D and 7B)

Proboscis very long, almost as long as haemocoel, occupying ~70% of haemocoel volume (Fig 6A: **pb**). Buccal mass occupying ~1/2 of haemocoel volume. Oral tube relatively long, broad, thickly muscular (**mj**), presenting several muscle layers in different dispositions (Fig 7B: **ot**). Differentiable jaws absent. Pair of dorsal folds of buccal cavity (**df**) very wide, with very narrow furrow separating folds in median line; dorsal folds becoming separated from each other at their middle level, showing series of narrow, longitudinal folds in this region, running in posterior direction longitudinally (Fig 7B: **ff**), flanked by pair of taller folds (**ef**), running up to esophageal gland. Odontophore muscles (Fig 6B and 6D): **m1d**) pair of narrow jugal muscles working as dorsal protractor muscles, relatively flattened, originating in anterior-dorsal surface of mouth, close to median line, running in posterior direction, inserting in posterior end of buccal mass; **mc**) buccal sphincter and circular muscles, relatively thin, inconspicuous; **mj**) jaws, oral tube and peri-buccal muscles, portion connected to odontophore working as odontophore protractors, inserting in posterior-ventral region of odontophore cartilages (Fig 6D: mj); **m2**) pair of broad dorsal retractor muscles of buccal mass, originating in ventral-posterior region of inner surface of haemocoel, originating in same of origin of pair of retractor muscle of proboscis (**rm**) (Fig 6A: **m2**), running towards anterior region, attached to lateral surface of esophagus, inserting in lateral-dorsal-posterior surface of buccal mass; **m3**) narrow and very thin pair of muscles, originating in lateral-ventral region of posterior odontophore surface, running dorsally, covering posterior region of odontophore, inserting in dorso-posterior region odontophore close to esophageal origin (Fig 6D: m3); **m4**) large pair of dorsal tensor muscle of radula, originating around origin of mj, broad at origin, running toward dorsum, covering entire inner odontophore structures and cartilages, inserting as two flaps, medial flap smaller (Fig 6D: **m4a**) connecting directly to radular ribbon, lateral flap broader (Fig 6D: **m4**), connecting laterally along subradular cartilage; **m5**) large pair of secondary dorsal tensor muscle of radula (Fig 6D: **m5**), originating in median-posterior region of m4, close to median line, running toward anterior region of odontophore for relatively short distance (1/3 odontophore length), inserting in shorter region of ventral surface of radular sac, just ventrally to insertion of m4; **m6**) horizontal muscle, relatively thin, connecting anterior-ventral margin of both odontophore cartilages for about 1/4 of their length; **m6a**) accessory horizontal muscle, same function and same size as m6, but located behind it at distance equivalent to half its length (Fig 6D: m6, m6a); **m7**) absent; **m10**) pair of ventral protractor muscle of buccal mass, originating in ventral and lateral regions around mouth, very broad and thick at origin, running toward posterior and medial regions of odontophore, becoming narrow and thin at insertion, inserting in postero-ventral level of odontophore (Fig 6B: **m10**); **m11**) narrow pair of ventral tensor muscles of radula (Fig 6D: **m11**) and auxiliary ventral protractor muscle of buccal mass (Fig 6A and 6B: **m11**), originating in middle ventral region of inner surface of haemocoel near origin of retractor muscle of proboscis (**rm**), running towards anterior region of odontophore, dorsally to rm, penetrating into ventro-posterior region of odontophore near radular sac (Fig 6B), running further anteriorly, flanking m6 and m6a, inserting in anterior end of radular ribbon; **m12, m13**) absent; **m14**) pair of accessory ventral protractor muscle of buccal mass, slightly thin, same origin as m10 but medial, running in posterior direction, covering m10, gradually becoming narrower, inserting in posterior limit of odontophore on m4 (Fig 6B). Pair of odontophore cartilages long, flattened, somewhat elliptical, most of muscles inserted on their outer surface (Fig 6D: **oc**). Radula long, about 3 times odontophore length (Fig 6A: **rs**), coiled behind buccal mass inside haemocoel.

#### Radula

(Fig 4A–4C): **rachidian** tooth ~1/6 of width of radular ribbon, central cusp broad, length ~1/2 of rachidian length, blunt, curved inwards, bearing pair of very low secondary cusps opposed to central cusp (Fig 4B); **lateral tooth** as wide as rachidian, flattened, curved inwards and medially, tip acute, carinated at periphery, slightly arched, base flattened, bearing very small secondary cusp at base of lateral edge of main cusp; **inner marginal tooth** similar to lateral tooth, but slightly narrower; **outer marginal tooth** similar to inner marginal tooth, but ~40% narrower and lacking lateral secondary cusp. Salivary glands clustering around anterior and middle esophagus, behind nerve ring (Fig 7B: **sg**), located at left-middle region of haemocoel (Fig 6A). Salivary ducts passing through nerve ring; penetrating in dorsal wall of buccal mass, running immersed along base of dorsal folds; opening at middle level of dorsal folds, near and bent toward median line (Fig 7B: **sa**). Anterior esophagus somewhat narrow, inner surface with pair of tall longitudinal folds (as extension of dorsal folds of buccal mass) (ef), flanking series of longitudinal folds (Fig 7B: **ff**). Middle esophagus broad, smooth; ventral esophageal gland massive, broad, well delimited, located in posterior-right region of haemocoel (Fig 6A: **ec**); inner surface of esophageal gland entirely filled with several thin septa, bearing fragile glandular tissue (Fig 7B: **ec**). Posterior esophagus narrow (Fig 6A and 6C: **es**), long (about half of total esophagus length), inner surface lacking folds. Posterior esophagus located inside visceral mass, gradually becoming broader, with smooth inner surface (Fig 6C). Stomach large (Fig 6C), u-shaped, located around penultimate whorl of visceral mass (Fig 6C: **st**); esophagus inserting on its left side with no clear distinction. Duct to digestive gland (Fig 6C: **dd**) located at transition between esophagus and stomach, almost as broad as esophagus, curved anteriorly, bearing series of transverse small folds along its ventral region; septa ending in duct bifurcation, at considerable distance from their origin. Intestine broad at origin, identifiable as right branch of stomach; running in anterior direction and leftward, parallel to posterior esophagus (Fig 6C: **in**); inner surface smooth. Digestive gland described above. Intestine, suddenly becoming bent rightward around left end of kidney, running attached to posterior surface of kidney along its entire length, crossing from left to right side of visceral mass anterior limit (Fig 6C). Rectum short, running along right margin of pallial cavity. Anus broad, siphoned, located close to anal siphon of mantle border (Figs 5B and 6C).

#### Genital system

Male. Testis running along columellar surface of visceral mass up to posterior limit of pallial cavity (Fig 5B: **ts**). Seminal vesicle intensely coiled, located in anterior projection of visceral mass, at left of esophagus, as extension of testis (Fig 5B: **sv**). Seminal vesicle with about same caliber along its length. Vas deferens suddenly running perpendicularly in anterior direction along approximately half of visceral mass width (Fig 5B: **vd),** in middle region of right surface of seminal vesicle mass; small aperture to sinus located in region preceding its exit to pallial cavity (Fig 7C: **vd**). Opened sperm grove beginning at its exit to pallial cavity floor (Fig 7C: **ps**), running up to penis base (Figs 5A and 7C), flanked by relatively thick, symmetrical edges. Penis long, with ~1/2 of head-foot length, almost cylindrical, curved distally (Figs 5A and 7C: **pe**). Penis groove running along its ventral surface up to penis tip. Penis tip bearing small, broad papilla. Penial gland located in middle region of right edge of penis (Fig 7C: **pl**), disform, occupying ~1/3 of penis surface.

Female (Fig 7A and 7D). Ovary occupying approximately same regions than testis of males, but broader posteriorly and very narrow anteriorly (Fig 7A: **oy**). Visceral oviduct (**vo**) running to right, in ventral surface of kidney. Visceral oviduct very slender, short, with ovarian tissue along its length (Fig 7A). In left half of visceral mass oviduct suddenly expanding, becoming thick glandular. Albumen gland of pallial oviduct (**ag**) with pair of glandular laminas, similarly sized, gradually increasing along its length. Bursa copulatrix (**bc**) balloon-shaped, located in middle level of pallial oviduct, insertion right; duct thick walled, as long as local pallial oviduct width, inner surface with 7–8 longitudinal simple folds (Fig 7D: **bc**). Bursa main region wide, with 4–5 spherical lobes, about as wide as local oviduct; walls thin, inner surface smooth (Fig 7D). Bursa orifice located just between both laminas of pallial oviduct. Female genital aperture (**ap**) as simple longitudinal slit, close to floor of pallial cavity far behind anus; edges thick.

#### Central nervous system

Nerve ring similar to those of remaining species. Degree of fusion of both cerebral ganglia high, almost resulting in single spherical mass. Cerebro-pedal and cerebro-pleural connectives very long, 5–6 times longer than both cerebral ganglia width.

#### Distribution

Known only from Trindade and Martim Vaz Islands.

#### Habitat

Rocks and corals, subtidal, ~20 m depth (Fig 3L and 3M).

#### Etymology

The specific epithet is in apposition; it is a reference to mammoths, which are widely recognized for their huge size and heavy build, an allusion to the large proportions and heavy shell of new species. Moreover, the long anterior projections of the shell are reminiscent of a mammoth's tusk. The epithet also follows the tendency of naming *Macrocypraea*’s after animals.

#### Measurements (respectively length, height, width in mm)

Holotype: 133.1 by 54.6 by 72.4; MZSP 108077: 95.8 by 46.7 by 54.9; MZSP 104754: 116.7; MZSP 108079: 74.1 by 32.6 by 41.1; MZSP 108084: 111.6; MZSP 108095: 114.9 by 56.6 by 65.9. MZSP 101404: 88.7 by 37.1 by 47.4; MZSP 108054: 103.3 by 46.7 by 55.3; MZSP 118452: 111.7 by 49.5 by 60.4. MZSP 121946: 120.1 by 53.1 by 65.0.

#### Material examined

Types. *Additional material*: BRAZIL. off Espírito Santo; Trindade Island, MNRJ 30770, 30771, 30772, 35142, 4 shells (Bruno Lobo col., 1916), Praia dos Cabritos (Cabritas), MNRJ not numb., 1 shell (A Coelho col., 31.x.1957), MNRJ 5055, 2 shells (L.S.Silva & J. Becker col., i.1966), MNRJ 5058, 3 shells (J. Becker col., xii.1958), MNRJ 5054, 1 shell (S. Ypiranga, A Coelho & J Becker col., x.1957), MZSP 108079, 1 subadult shell, 20°29’38.2”S 29°19’50.2”W, 10 m (J.B. Mendonça col., 12.vi.2012), MZSP 108054, 1 shell (J.B. Mendonça coll., 17/vi/2012), Praia da Tartaruga, MNRJ 5057, 3 shells (J. Becker col., xii.1958), MZSP 101404, 1 shell+ 1 fragment (D. Abbate & P.O. Lima col., 25.vii.2011), MZSP 121241, 1 shell fragment (Simone et al col., 25.vii.2011), MZSP 104152, 1 subadult shell fragment (C.H. Guimarães col., 27.ii.2012), Calheta, MNRJ 5060, 3 shells (B. Prazeres col., xii.1975-ii.1976), near local lighthouse, 20°29′52.3″S 29°19′15.6″W, 12.9 m, MZSP 118452, 1 shell (J.B. Mendonça coll., 17/iv/2012), 20°30’26.1”S 29°18’44.2”W, MZSP 108095, 1 shell (J.B. Mendonça col.), Praia do Monumento, 20°30’10.3”S 29°20’36.1”W, 15 m, MZSP 121496, 1 shell (J.B. Mendonça col., 2.xi.2014), Ponta Norte, 20°29’18.7”S 29°20’18.3”W, 10 m, MZSP 108078, 1 shell, MZSP 121237, 1 shell fragment (J.B. Mendonça coll., 03/vii/2012), between Calheta and Andradas Beach, 20°30’37.9”S 29°18’32.0”W, 10 m MZSP 104754, 1 fragmented shell, (C.H. Guimarães col., 14.ii.2012); Parcel das Tartarugas, 20°31’01.3”S 29°17’56.9”W, 5 m, MZSP 108084, 1 fragmented shell depth (J.B. Mendonça col., 11.vi.2012), Enseada Noroeste, 20°29’40.9”S 29°20’44.1”W, 12.2 m, MZSP 104744, 2 shell fragments (C.H. Guimarães col., 30.i.2012); Martim Vaz, 20°28’28.42”S 28°51’24.56”W, 21 m, 1 shell (J.B. Mendonça col., 21.vi.2015).

### *Macrocypraea cervus* (Linnaeus, 1771)

**Fig 8:**
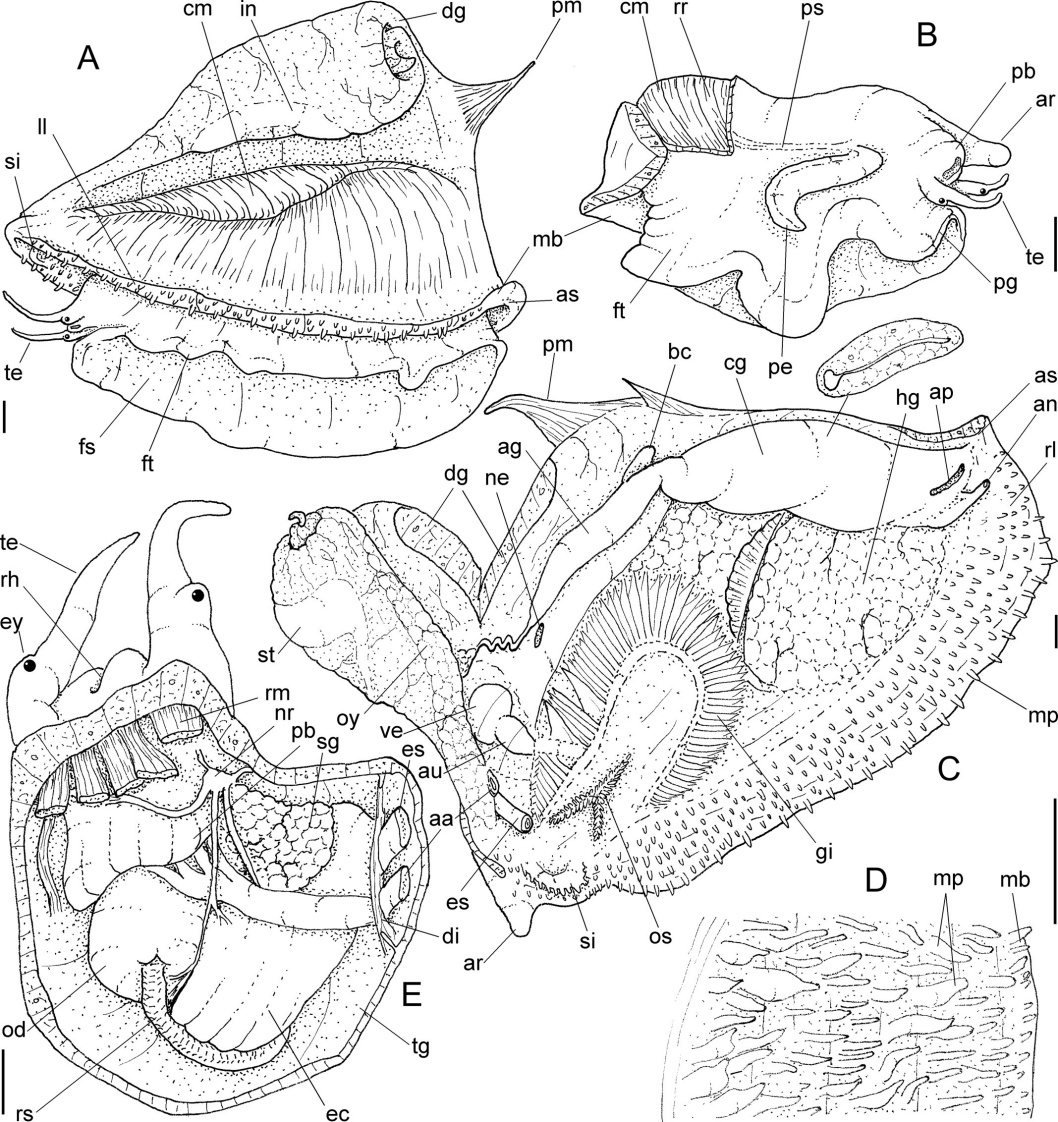
*Macrocypraea cervus* anatomy. (A) Whole specimen extracted from shell, left view, visceral mass slightly deflected upwards. (B) Head-foot, male, right view.; (C) Pallial roof and visceral mass, ventral view, portion of gill ventral to pericardium removed, anterior region of digestive gland (dg) and hypobranchial gland (HG) partially sectioned transversally, transverse section of indicated region of capsule gland (cg) also shown. (D) Right mantle lobe, middle region, outer view. (E) Head, ventral view, foot and columellar muscle removed. Scales = 5 mm.

**Fig 9:**
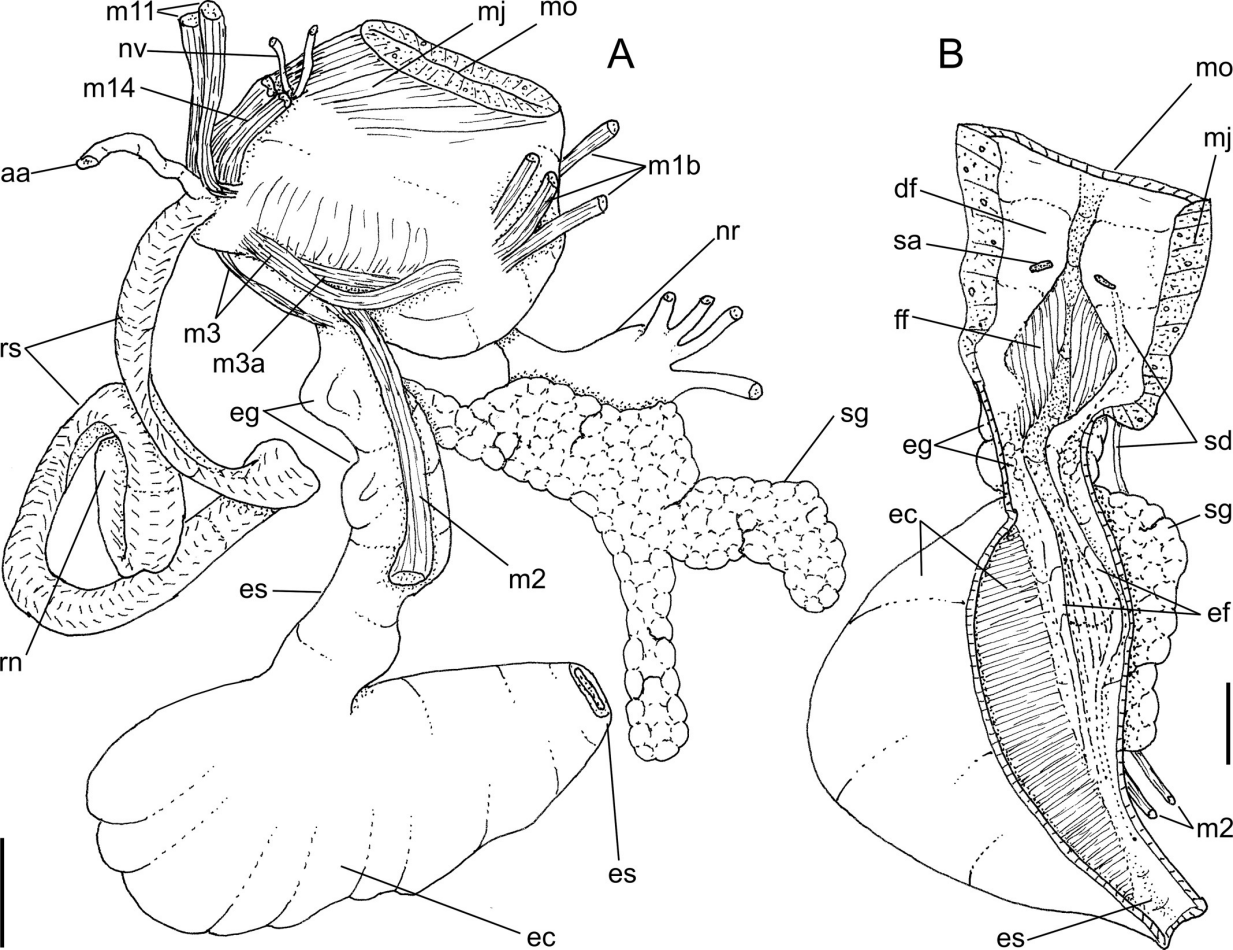
*Macrocypraea cervus* anatomy. (A) Foregut, left view, some structures deflected, (B) Same, ventral view, opened longitudinally, odontophore removed. Scales = 5 mm.

**Fig 10:**
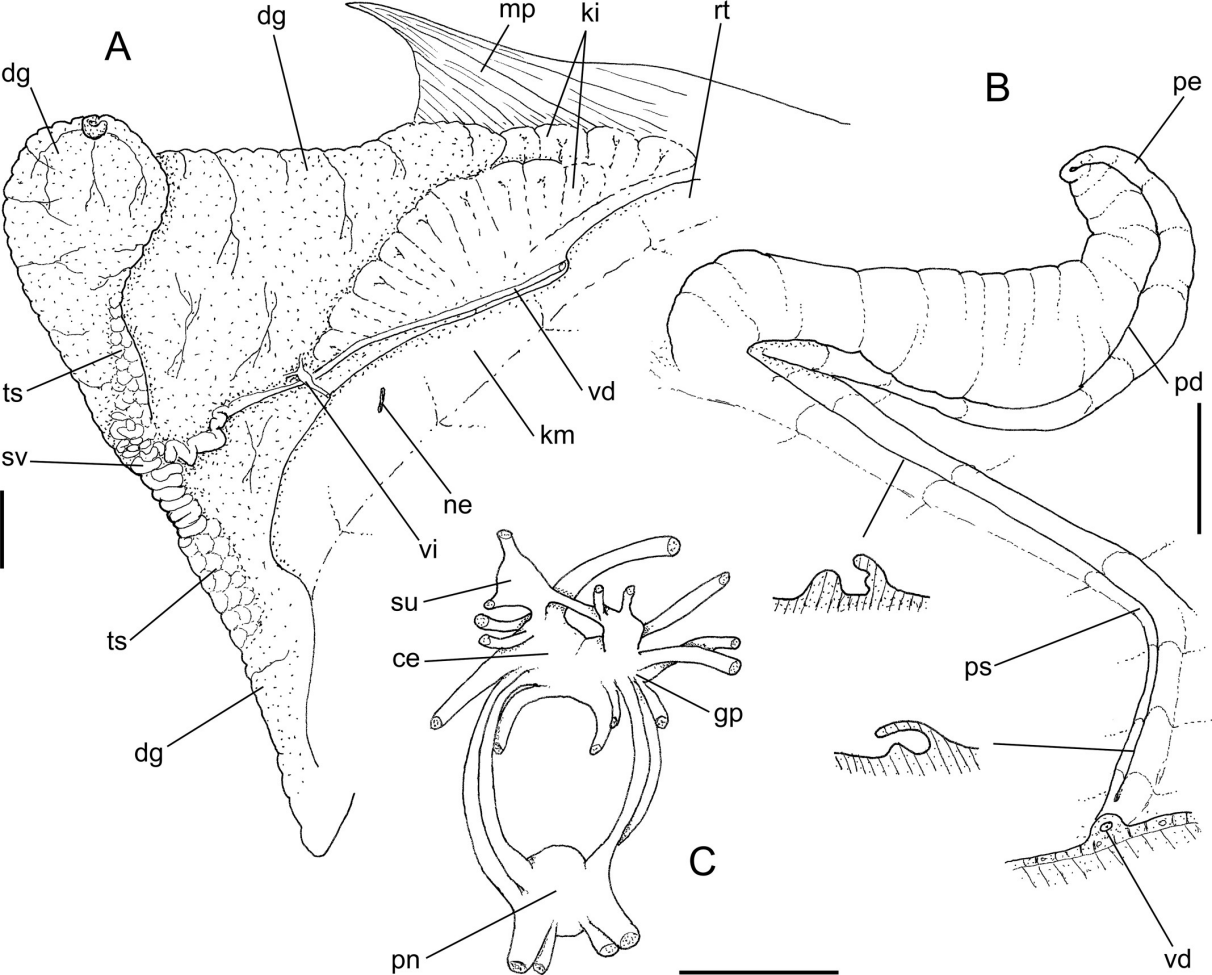
*Macrocypraea cervus* anatomy. (A) Visceral mass, male, ventral view, partially uncoiled. (B) Right region of pallial cavity floor and penis, dorsal-slightly posterior view, transverse section in two indicated regions also shown. (C) Central nervous system, ventral view. Scales = 5 mm.

**Fig 11:**
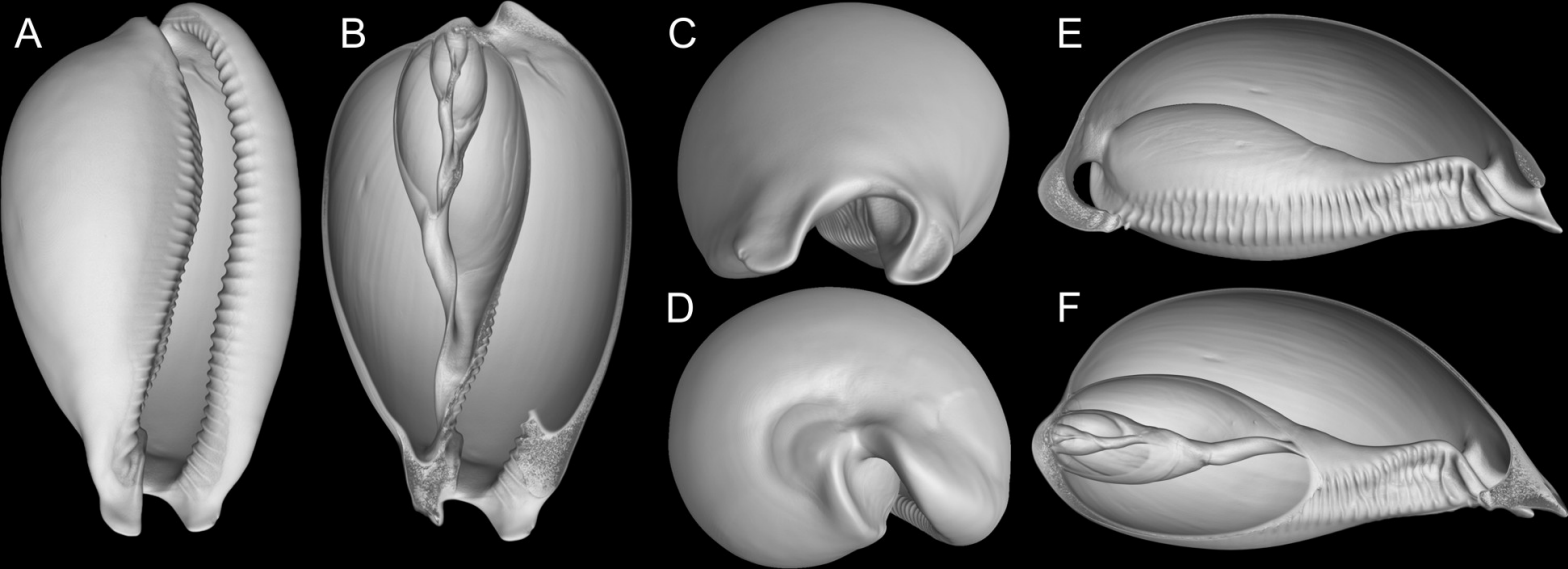
*Macrocypraea cervus*, 3D reconstructions of shell (CT-Scan) MZSP 121238 (L 98.5 mm). (A) Frontal view. (B) Same, more ventral portions removed. (C) Anterior view.(D) Posterior view. (E), Right view, right half removed. (F) Same, longitudinal section along columellar axis.

Figs 3G–3K, 3N–3P, 4D–4E, 8–10 and 11–11F

Synonymy see [2]. Complement:

*Cypraea cervus* Linnaeus, 1771[15]: 548.

*Macrocypraea cervus*: Meyer, 2003[16]: 411; Soriano, 2006[17]: 49, Table 3; Rosenberg et al., 2009[18]: 636; Lorenz, 2017[2]: 287, text figure., pl. 58.

*Macrocypraea (Lorenzicypraea) cervus*: Petuch & Drolshagen, 2011[19]: 121–122.

#### Type locality

Not stated.

#### Diagnosis

Shell large (~120 mm), with very numerous minute whitish spots, never ocellated, aperture widening anteriorly. Mantle lobes papillae small, simple, slender, very numerous. Hypobranchial gland very thick. Osphradium small, with branches very narrow. Kidney with lobe wide, with radial arrangement. Odontophore pair m3 twofold (m3, m3a). Anterior esophagus with distinct gland. Pallial spermoduct with edges strongly asymmetrical. Albumen gland clearly narrower than capsule gland. Bursa copulatrix simple, curved.

### Distinctive description

#### Shell (Figs 3G–3K, 3N–3P and 11A–11F)

Shell description in Lorenz ([2]: 287). Pair of anterior projections flanking canal ~1/8 of shell length (Fig 11A and 11C). Inner lip slope with teeth relatively short, perpendicular to longitudinal axis (Fig 11E); anterior concavity preceding canal relatively small (~1/3 of local shell height), lacking ventral nodules (Fig 11D and 11F). Left edge of canal oblique (Fig 11E).

#### Head-foot (Fig 8A and 8B)

Characters similar to *M*. *mammoth*. Distinctions as follows: Cephalic tentacles more ventrally positioned. Pairs of retractor muscles of proboscis (Fig 8E: rm) of equivalent size. Proboscis much shorter (described below).

#### Mantle organs (Fig 8C and 8D)

Similar to *M*. *mammoth*. Distinctions and remarks as follows: Outer surface of exposed part of mantle dark brown, bearing many relatively small, somewhat uniformly distributed papillae (mp). Each papilla mostly cylindrical, distal end tapering up into sharp pointed tip (Fig 8D: mp), base rarely narrower than middle region; profile broad, rather cylindrical. Siphon (si) bearing series of small papillae along its margin. Osphradium (os) much smaller, occupying ~1/30 of pallial roof area, widely separated from siphon and gill. Osphradium with three branches of roughly similar size and shape, each encompassing ~1/2 of total osphradium length. Each gill filament taller, narrower, triangular, tip sharper (Fig 8D: gi). Distance between gill and visceral mass very close to kidney in posterior half, suddenly becoming far from kidney in anterior half. Hypobranchial gland thicker (hg).

#### Visceral mass (Fig 8A, 8C and 10A)

Same characters as *M*. *mammoth*.

#### Circulatory and excretory systems (Fig 10A)

Similar to *M*. *mammoth*.

#### Digestive system (Figs 9B–10A)

Most attributes similar to *M*. *mammoth*. Distinctions and notable features as follow: Proboscis much shorter (Fig 8E: pb), length ~1/3 of haemocoel length, volume ~30% of haemocoel volume. Buccal mass occupying ~1/4 of haemocoel volume, protruding beyond proboscis. Pair of dorsal folds of buccal cavity (df) slightly narrower; dorsal folds becoming narrow, separated from each other in their middle level, space between folds bearing two series of narrow, slightly oblique smaller folds (Fig 9B: ff) delimited by pair of taller folds (ef) as continuation from buccal dorsal folds, running along entire middle esophagus, fading in posterior third of esophageal gland (Fig 9B). Odontophore muscles (Fig 9A) also similar, differing by **m1b**) composed of three pairs of narrow jugal muscles, functioning as dorsal protractor muscles, relatively flattened, originating on latero-dorsal surface of mouth, extending postero-dorsally, inserting into dorso-lateral outer wall of buccal mass; **m4d**) absent; **m3**) similar characters but twofold (Fig 9A: m3 + m3a), with inner pair (m3a) smaller and thinner; **m10**, **m11**, **m14**) pairs of similar features, but much narrower and thinner. Radular teeth (Fig 4D and 4E) also similar, differences as follows: **rachidian** tooth ~30% narrower, terminal cusp curved forwards (instead of inwards); **lateral tooth** with sharper pointed tip, straight (instead of undulated); **both marginal teeth** also similar, straight, with sharper pointed tip. Salivary glands slightly larger (Fig 9A: sg). Salivary ducts also opening in middle level of dorsal folds, but positioned more laterally, at middle of dorsal fold width (Fig 9A; sa). Dorsal folds flanking pair of sets of longitudinal folds (Fig 9B: ff), ending before anterior esophagus. Anterior esophagus bearing lobes of glandular tissue (Fig 9A and 9B: eg); inner surface with a pair of tall longitudinal folds (extension of dorsal folds of buccal mass) (ef). Middle esophagus with pair of folds (ef) coming from anterior esophagus, running along large esophageal gland aperture (ec); this pair of folds fading before posterior region of esophagus. Stomach large (Fig 8C), u-shaped, located about in penultimate whorl of visceral mass (Fig 8C: st); esophagus inserting into its left side with no clear distinction. Mid and hindgut (Fig 8C) similar to preceding species. Anus narrow, siphoned (Fig 8C: an).

#### Genital system

Male. Similar to *M*. *mammoth*, except for: branch of testis at left of seminal vesicle (Fig 10A: inferior ts); Seminal vesicle (Fig 10A: sv) coiled in T-fashion, with its left coils slightly broader than the right ones. Opened sperm grove (Figs 8B and 10B: ps), flanked by relatively thick edges, with left edge narrower and taller than right edge mainly in its posterior region. Penis (Figs 8B and 10B: pe) slightly broader, lacking penial gland in its middle region.

Female (Fig 8C). Features similar to *M*. *mammoth*. Notable features as follows: Visceral oviduct slightly more convolute. Albumen gland of pallial oviduct (ag) ~1/3 of capsule gland width, with similar length. Bursa copulatrix (bc) also balloon-shaped, but narrower and simpler, composed of a single blind-sac structure bent to the right; inner surface bearing 7–8 tall longitudinal folds.

#### Central nervous system (Fig 10C)

Cerebral (ce) and pedal (pn) ganglia very close to their counterparts, commissure indistinct. Pair of cerebral ganglia occupying ~1/40 of haemocoel volume. Pleural ganglia (gp) partially fused to cerebral ganglia, with indistinct limits. Supra and suboesophageal ganglia (su) located dorsally, each connected to adjacent cerebral ganglia, size ~1/4 of cerebral ganglion size, bearing connective between them as wide as their own width. Pair of cerebro-pedal and pleuro-pedal connectives relatively symmetrical, long, their length ~twice cerebral ganglia width. Pair of pedal ganglia (pn) ~70% of cerebral ganglia size, almost spherical.

#### Distribution

South Florida to north of Cuba.

#### Habitat

Rocks and corals, subtidal, 0–53 m [18].

#### Material examined

USA: **Florida**; Florida Keys, off Marathon (E. Malone col., 1970), MZSP 121238, 1 shell, MZSP 121239, 1 shell, MZSP121240, 1 shell (ex. Colella collection), SE point of Big Pine Key, ANSP 93276 (A6307), 1♀ (H.A. Pilsbry col., 1907, ex C.B. Moore), Bahia Hondo Key, 24°40’04”N 81°15’55”W, ANSP 216746 (A15658), 1♂ (A.J. Ostheimer col., 2.vi.1958); Indian River, off Wabasso, sand flat north of rest area on causeway state route 512, 27°45.943’N, 80°25.257’W, MZSP 46866, 1 shell (Simone col., 5/VIII/2004); Keys, N of Keys Marine Laboratory, 24°49.78’N 80°48.51’W, MZSP 36106, 1♂, 2♀ (Simone col., 22.vii.2002). CUBA: **Cienfuegos** Province; off Cienfuegos city, 2–4 m, MZSP 115362, 1 shell (under dead corals, local people col., vi.2000, ex Femorale). MEXICO: Gulf of Mexico, MZSP 8149, 1 shell (Sowerby col., 1896). COSTA RICA: Western Coast, Tamarindo Bay, left of Hotel Rocky Point. 17°N, 85°20’W, MZSP 131503, 1 shell (B. Cook coll., 1/ii/1982).

#### Measurements (height, length, width in mm)

MZSP 8149: 64.1 by 136.3 by 80.0; MZSP 46866: 36.5 by 71.4 by 41.0; MZSP 115362: 44.2 by 92.2 by 55.5; MZSP 121238: 47.7 by 98.5 by 57.2; MZSP 121239: 44.2 by 95.5 by 55.4; MZSP 121240: 55.7 by 116.2 by 67.7.

#### Remarks

*Macrocypraea cervus* also has some anatomical exclusivities that have been explored in the present comparative description. Some are particularly interesting, e.g., the slender, small, and more numerous papillae of the mantle lobes (Fig 8C and 8D: mp), the thick hypobranchial gland (Fig 8C: hg) reinforced by wide transverse septa, and the very narrow osphradium branches (Fig 8C: os). The wide renal tissue clearly organized in radial folds (Fig 10A: ki), the two-folded odontophore pairs m3 (Fig 9A: m3, m3a), the clear, bulging glandular region of the anterior esophagus (Fig 9A and 9B: eg), the strong asymmetry of pallial spermoduct edges (Fig 10B: ps), mainly in its posterior region, the clear distinction of width between the albumen and capsule glands in the pallial oviduct (Fig 8C: ag, cg), and the bursa copulatrix comprised of a simple, bent sac (Fig 8C: bc) are also noteworthy exceptionalities.

### *Macrocypraea cervinetta* (Kiener, 1843)

Fig 12A–12F

**Fig 12:**
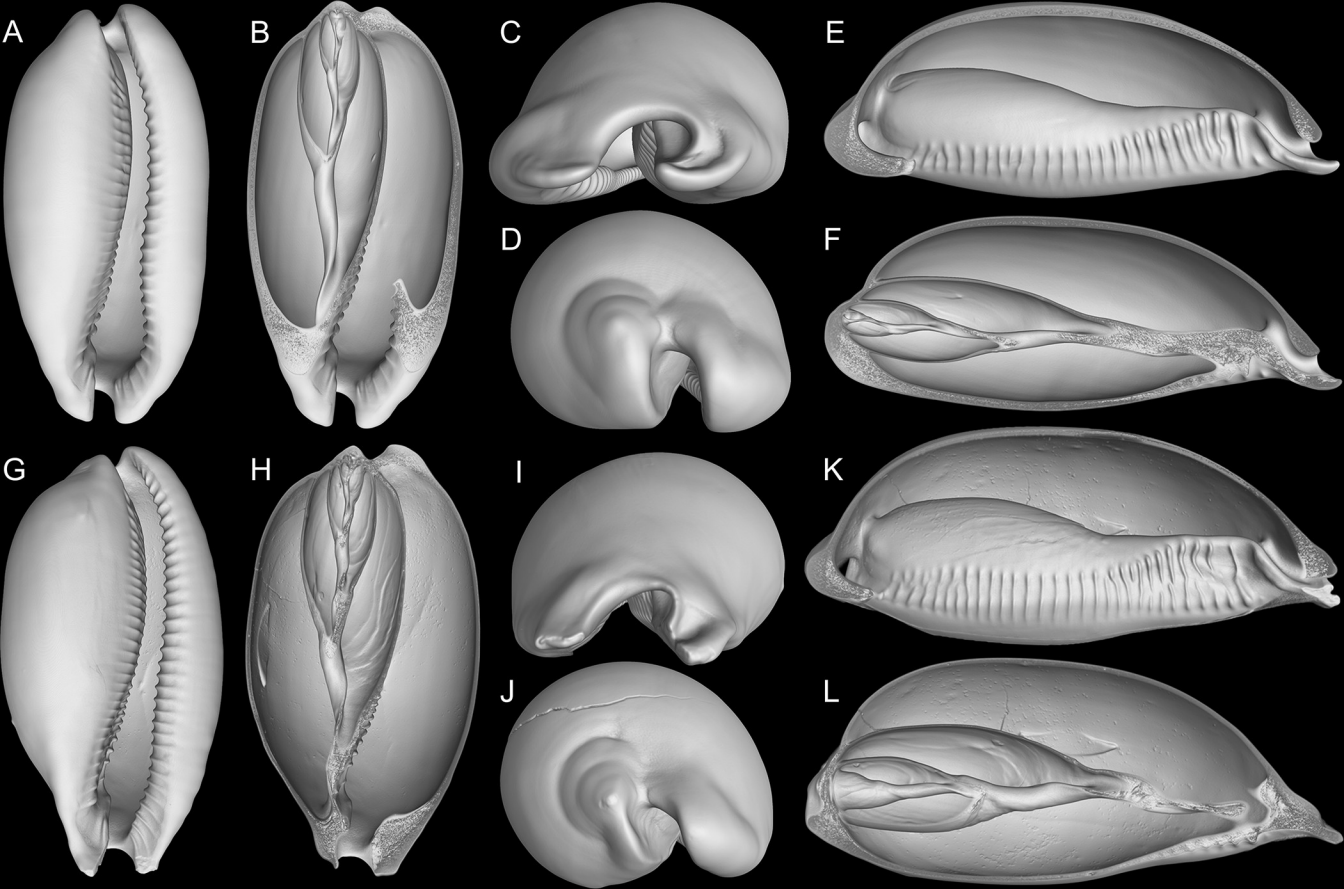
*Macrocypraea*, 3D reconstructions of shells (CT-Scan). (A-F) *M*. *cervinetta* MZSP 131503 (L 58 mm). (A) Frontal view. (B) Same, more ventral portions removed. (C) Anterior view; (D) Posterior view. (E) Right view, right half removed. (F) Same, longitudinal section along columellar axis. (G-L) *M*. *zebra* MZSP 38946 (L 100 mm). (G) Frontal view. (H) Same, more ventral portions removed. (I) Anterior view. (J) Posterior view. (K), Right view, right half removed. (L) Same, longitudinal section along columellar axis.

Synonymy see [3]. Complement:

*Cypraea cervinetta* Kiener, 1843: pl. 6, Figs 1 and 2.

*Macrocypraea cervinetta*: Meyer, 2003[16]: 411; 2004[20]: 135; Simone, 2004[3]: 39, Figs 5 and 6, 71, 144–153; Paulay & Meyer, 2006[21]: 273, Table 2; Soriano, 2006[22]: 49, Table 3; Guevara-Fletcher et al., 2011[17]: 8, Table 5; Torreblanca-Ramírez et al., 2012[23]: 286, Table 1; Landa-Jaime et al., 2013[24]: 1125, Table 1; Torreblanca-Ramírez et al., 2014[25]: 555, Table 1, Fig 2J(17); Lorenz, 2017[2]: 289, text. figure., pl. 60; Torreblanca-Ramírez et al., 2017[26]: 86, Table 1, Fig 2(52).

#### Distribution

Gulf of California, Pacific Central to South America.

#### Material examined

As in [3].

#### Measurements

As in [3].

#### Remarks

The only representative of the genus in the Pacific. Conchologically, it can be readily distinguished from the remaining species by the marked anterior widening of the aperture (Fig 12A and 12B). Most other shell characters such as color, width/height ratio, and general outline overlap with *M*. *zebra* in varying degrees. The pair of anterior projections flanking canal relatively short (~1/12 of shell length; Fig 12B and 12C), left edge oblique. Inner lip slope with relatively short, spaces teeth, mostly positioned perpendicular to longitudinal axis (Fig 12E). Anatomically, it differs from the remaining species of the genus in having a more anterior origin of the auricle; kidney with ventral lobe bearing a mosaic of pores instead of longitudinal folds; narrower rachidian tooth; narrower seminal vesicle; and a straighter penis with a rounder tip (Simone, 2004). Simone (2004) reported no significant anatomical differences between individuals from the Gulf of California (i.e., *M*. *cervinetta californica*) and the Central-South America (nominal subspecies). Conchological distinctions between the two subspecies were discussed by Lorenz (2017: 290).

### *Macrocypraea zebra* (Linnaeus, 1771)

Fig 12G–12L

Synonymy see [3]. Complement:

*Macrocypraea zebra*: Meyer, 2003[16]: 411; Simone, 2004[3]: 35, Figs 1–4, 70, 116–143; Soriano, 2006[17]: 49, Table 3; Rosenberg et al, 2009[18]: 636; Rios, 2009[14]: 132, text figure. Lorenz, 2017[2]: 288, text figure.

*Macrocypraea zebra zebra*: Lorenz, 2017[2], 288, text figure.

*Macrocypraea zebra dissimilis*: Lorenz, 2017[2]: 289, text figure.

#### Distribution

North Carolina to southern Brazil.

#### Habitat

Rocks and corals, subtidal, 0–37 m depth [3].

#### Material examined

Specimens of *Macrocypraea zebra* listed in Simone [3], with the addition of the following: DOMINICAN REPUBLIC: Puerto Plata Province; Puerto Plata, 2–3 m depth MZSP 115363, 1 shell (local people coll., XI/2011). HAITI: Nippes Department; Anse-à-Veau Commune, MZSP 115364, 1 shell (local people coll., 1986). BRAZIL: Bahia State; reefs off Alcobaça, 20–25 m depth, MZSP 72653, 20 shells (A. Bordart coll., VII/2004). Rio de Janeiro State; Arraial do Cabo, 1–2 m, MZSP 73553, 8 shells (P. Gonçalves coll., XII/2003). São Paulo State; Ilhabela, 23°46’S, 45°21’W, MZSP 121109, 20 shells (J. Colella coll., VII/1968). BRAZIL: Santa Catarina State; precise locality unknown, MZSP 38946, 1 shell (De Fiore coll., date unknown).

#### Measurements (mean values ± standard deviation, in mm; n = 50)

H = 26 ± 7, L = 64.5 ± 16.9, W = 33.7 ± 8.7.

#### Remarks

Based on the specimens analyzed by Simone [3] and in the present samples, there are no significant anatomical or conchological differences between individuals of the Caribbean (i.e., *M*. *zebra zebra*) and Brazilian (*M*. *zebra dissimilis*) populations. As such, there are not enough characters to distinguish these subspecies other than the supposed range gap. Lorenz [2] mentioned that the division is corroborated by molecular data, bud did not mention any reference or study in progress and provided no additional evidence to endorse the separation. In our understanding, these subspecies must remain invalid until further evidence is presented. The anterior pair of projections franking canal is slightly longer than that of *M*. *cervinetta*, but shorter than that of *M*. *mammoth*, about 1/10 of shell length (Fig 12G and 12I). The inner lip slope has fewer and shorter teeth than *M*. *mammoth* (Fig 12K), disposed perpendicularly to the longitudinal shell axis; those teeth from anterior concave region are interrupted at middle, with weak ventral nodes (Fig 12K). The left edge of canal is more uniform and arched, oblique positioned (Fig 12K and 12L).

## Discussion

Individuals of *Macrocypraea mammoth* have been reported from Trindade and referred to as *M*. *zebra* or its older combination *Cypraea zebra* in the literature and museum collections for quite a long time. Even so, most of the specimens deposited in museum collections consist of fragmentary and empty shells. The oldest lots, which are deposited in the Museu Nacional do Rio de Janeiro (MNRJ), date back to 1916. Leal [13] had already pointed out the large size of this species as distinctive from the continental specimens, regarding it as conspicuous and abundant among beach drift in Trindade. By the time Leal reported his findings, however, no other specimens had been collected in the island since 1976, raising the possibility of extinction. The lack of living specimens and the fact that most of the recovered shells were badly damaged did nothing but reinforce this suspicion. Still, the live individuals collected in the latest samplings nearly a hundred years since the first shells were found at Trindade dismissed this suspicion. They also revealed crucial anatomical information that further distinguished the newly introduced *M*. *mammoth* from its ally species, *M*. *zebra*. Some of the samples studied herein with precise collection data were obtained during the daytime, since nocturnal diving in the region is forbidden by the Brazilian maritime authority. It is widely known that related species such as *M*. *zebra* are mostly active during the night [27], and we believe the same could be assumed for *M*. *mammoth*. The lack of nocturnal samplings may thus help explain why live specimens were never collected in the past.

Conchologically, *Macrocypraea mammoth* is immediately distinguished from the remaining species by its proportionally much heavier shell (Fig 4G, 4J and 4K), with thick walls. From *M*. *zebra* it differs by its wider and more rounded outline (Fig 4F and 4J), and color pattern with proportionally larger beige spots. It differs from *M*. *cervus* by having a slightly more elongated outline, with a more tapered posterior end (both laterally and dorso-ventrally, Fig 4H and 4K), fewer teeth on the outer lip, and by a color pattern with larger, less coalescent beige spots, the most basal of which can present a dark brown (ocellated) central area. Moreover, *M*. *mammoth* differs from *M*. *cervinetta* in having a much larger and heavier shell, more inflated outline, more pointed anterior projections flanking the siphon area, and more spaced apertural teeth. *M*. *mammoth* can be further told apart from both other Western Atlantic cypraeid species by having a longer tapered posterior end and a more inflated base (Figs 2A, 3F and 4F–4K). Additionally, the shell of *M*. *mammoth* has a longer anterior and more flattened anterior end than those of the remaining congeners; the anterior, siphonal region actually looks like a pair of small spatulas laterally flanking the siphon, projected anteriorly. The closest looking species regarding this character is *M*. *zebra*, but this pair of anterior projections are shorter and the right one is slightly swollen. Differences in the average number of labial teeth between of the Western Atlantic *Macrocypraea* had already been reported, with *M*. *cervus* showing 15% more teeth on the outer lip than *M*. *zebra* [27]. *M*. *mammoth*, is similar to *M*. *zebra* in this regard, having an equivalent number of columellar and outer lip teeth.

*Macrocypraea zebra* was so far the single species of the genus that occurred in Brazilian coast. It has 11 nominal synonyms usually found in literature (e.g., [3, 28, 29]), all them described for the North Atlantic and Caribbean. Some synonyms were described without stated locality (*Cypraea dubia* Gmelin, 1791; *C*. *clauca* Röding, 1789; *C*. *dissimilis* Schilder, 1924), with no indication of they are based on South Atlantic specimens. Those that were figured show more elongated shells that are comparable to *M*. *zebra*, rather than *M*. *mammoth*. Therefore, no synonym of *M*. *zebra* can be revalidated in order to designate the new species from Trindade.

The CT-Scan examination of the four *Macrocypraea* species (Figs 4, 11 and 12) revealed further shell differences. *M*. *mammoth* has the thicker shell walls (Fig 4G, 4J and 4K), which is common in gastropods living in oceanic islands (personal observation), which is possibly an adaptation against the local parrotfishes (Scaridae). The inner slope of the inner lip is particularly informative, as *M*. *mammoth* has radially, more densely arranged, and longer teeth (Fig 4J), while the remaining species have the shell region has shorter and more spaced teeth, arranged perpendicularly to the longitudinal shell axis (Fig 11E, Fig 12E and 12K). The density of teeth of *M*. *mammoth* is only comparable to *M*. *cervus* (Fig 11E). The anterior concavity preceding the canal is larger in *M*. *mammoth* (Fig 4J) than the remaining species (Figs 11E, 11F, 12E and 12K), and has ventral nodes at the end of the teeth, which are absent in the other species. The highly developed inner lip slope is visible in apertural view (Figs 2B, 2H, 2J, 3B and 4F), a region that remains hidden in the other species (Figs 11A, 12A and 12G). The inner shape of the spire is more spherical in *M*. *cervus* (Fig 11B and 11F), while it is elongated in the remaining species (Figs 4G, 4K, 12B, 12F, 12H and 12L). The left edge of the anal canal usually is thick in *M*. *cervinetta* (Fig 12D) and in *M*. *mammoth* (Fig 4I), while it is a narrow fold in *M*. *zebra* (Fig 12J) and in *M*. *cervus* (Fig 11D).

Regarding the fossil species of *Macrocypraea* [19, 30–32], *M*. *mammoth* can be distinguished from *M*. *veintensis* Perilliat, Avendano & Vega, 2003 (Eocene, Mexico), in having a much larger shell (that species is about 40 mm), wider aperture, not so rounded outline, slightly more projected anterior region, and fewer apertural teeth. It differs from *M*. *spengleri* Petuch, 1990 (Figs 5 and 6 in [31]) and from *M*. *joanneae* Petuch, 2004 (Fig 8.1 in [31]) (both Pleistocene, Florida) in lacking an inflated shell, with widely rounded posterior region, narrower anterior region of the aperture, in having fewer apertural teeth, and by the less truncated anterior region; from *M*. *joanneae*, in particular, *M*. *mammoth* still differs in having a much more bulged posterior region of the shell, mainly on its left side; and from *M*. *spengleri*, in particular, in having a narrower aperture, mainly anteriorly. It differs from *M*. *anguillana* (Cooke, 1919) (Miocene, Anguilla) in being larger (that species is about 30 mm) and having a more visible spire. From *M*. *trinidadensis* (L Miocene, Trinidad) by the larger size and by the slightly wider outline.

Anatomically, *M*. *mammoth* can be easily distinguished from the remaining species through the set of characters listed in its diagnosis. Notably, the presence of pallial papillae bearing 3–5 small aligned terminal projections (Fig 5B: mp), as opposed to a single terminal projection in the remaining species. Remarkably, the osphradium in *M*. *mammoth* looks somewhat deformed, with a shortened posterior branch as if it were severed, and an elongated and slightly narrower anterior branch (Fig 5B: os). This bizarre osphradium topology was found in all *M*. *mammoth* specimens examined herein. The pair of odontophore dorsal protractor muscles (Fig 6B: m1d) also looks exclusive since the remaining species have different arrangements. In *M*. *cervinetta*, the arrangement is somewhat similar (Fig 149: m1d in [3]), but much smaller, while in *M*. *zebra* (Figs 123, 125: m1b in [3]) and *M*. *cervus* (Fig 9A), they comprise several small pairs branched far from the median line. The pair of odontophore ventral protractor muscles of *M*. *mammoth*, on the other hand, is different in being rather lateralized and bearing 3–4 separated branches (Fig 6B: m10). The folded portion of inner surface of the esophageal origin (Fig 7B: ff) is a single set of folds, running along the anterior esophagus, while this folded region is normally separated into a pair of triangular region on posterior half of the dorsal folds of buccal cavity (Fig 124: ff in [3]) (Fig 9B: ff). The middle esophagus lacking longitudinal folds flanking the esophageal gland (ec) is also exclusive (Fig 7B: es), which is normally found in the other species (Fig 124: es in [3]) (Fig 9B). The seminal vesicle of *M*. *mammoth* is also very irregularly coiled, as usual for *Macrocypraea*, however, the structure looks with the same width along its length (Fig 10A: sv), while those of the other congeneric species have two clearly uniform regions, a left narrow one, and a wide right one (e.g., Fig 10A: sv). The pallial sperm duct (Fig 7C: ps) is flanked by similar-sized folds, while in the other species an asymmetry is present. The penis of *M*. *mammoth* is somewhat as usual for *Macrocypraea*–an elongated, simple structure curved at the tip. However, it has an exclusive well-developed glandular region, bulging in a side of middle penial region (Fig 7C: pl). The bursa copulatrix of *M*. *mammoth* is also different in having a long duct and a single wide, thin-walled region at tip (Fig 7D: bc), while in the remaining species the bursa is strongly bent, with a short or absent duct, and bearing strong inner folds (Figs 143, 153: bc in [3]) (Fig 8C: bc).

With the descriptions of *Macrocypraea mammoth* and *M*. *cervus*, the anatomy of every extant species of *Macrocypraea* has now been described in detail. Even so, the anatomical diagnosis of the taxon remained unchanged. Nevertheless, adding the present data to the morphology-based phylogeny by Simone [3], which already contained *M*. *zebra* and *M*. *cervinetta*, reinforces the monophyly of *Macrocypraea*. The main characters are: 1) osphradium separated from posterior region of the gill; 2) pair of odontophore muscles mc comprising two bundles; 3) pair of m11 with insertion surrounding ventral region of radular sac base; 4) bursa copulatrix located at middle level of pallial oviduct. Another set of character are present in remaining tree species excepting *M*. *cervus*, as follows: 1) pallial papillae stubby, broad, with narrow base; 2) odontophore pair m7 originating at anterior border of ventral m4 branch; 3) bursa copulatrix U-shaped, bearing inner folds. A close relationship of *Macrocypraea* with *Cypraea* s.s. was obtained by morphological phylogenetic approach [3], and it was somewhat corroborated by molecular approach (Fig 4 in [16]; Fig 2 in [20]), although other two genera not included in the morphological study resulted still closer–*Leporicypraea* Iredale, 1930, and *Mauritia* Troschel, 1863.

The discovery of a new cypraeid species is an uncommon event in recent times and serves two immediate purposes. First, it is as a reminder of how poorly known the Brazilian mollusk fauna is, particularly in remote areas such as oceanic islands. Second and most importantly, it draws attention to the necessity of protecting these animals, which, because of the endemicity and rarity, may become endangered in the near future.
