# Supplementary material for: A new species of Macrocypraea (Gastropoda, Cypraeidae) from Trindade Island, Brazil, including phenotypic differentiation from remaining congeneric species
Source: PLoS One. 2020 Jan 8;15(1):e0225963. doi: 10.1371/journal.pone.0225963 (PMC6948752; doi:10.1371/journal.pone.0225963)
Supplement: Attachment — Submitted filename: Rebuttal letter Macrocypraea2.pdf [file pone.0225963.s001.pdf]

Rebuttal Letter

The present paper PONE-D-19-30595 is a corrected and improved version of the PONE-D-19-26676. According to the email Nov 5/2019, this rebuttal letter is necessary. To attend the request, I reproduce below the decision email sent by Dr. Vermeij, in such I answer every comment and suggestion IN RED done by the referee Dr. Gary Rosenberg. So, all red texts are inserted on the original email. After the answered email, a version of the new version of the paper with all modifications showed is presented, all changes are in tracking mode.

PLOS ONE Decision: PONE-D-19-26676 - [EMID:66ad3b424b70cf58]

PLOS ONE <em@editorialmanager.com> qua, 23 de out 05:42 (há 5 dias)

ONE-D-19-26676
Revisiting the systematics of Macrocypraea (Gastropoda, Cypraeidae), with description of a new species from Trindade Island, Brazil
PLOS ONE

Dear Dr. Simone,

Thank you for submitting your manuscript to PLOS ONE. After careful consideration, we have decided that your manuscript does not meet our criteria for publication and must therefore be rejected.

Specifically:

The single outside review I was able to obtain is very thorough and raises important questions about the phylogenetic analysis. In my own reading of the paper, I would concur with the reviewer. I find the anatomical description adequate, and I concur with the authors that M. rhinoceros is a valid species. The best course of action, it seems to me, is to publish the new species and its anatomy*, but to omit the phylogenetic analysis*. I agree with the reviewer that the fossil taxa need to be discussed* in any published form of the present manuscript. I also urge the authors to read and eventually to cite Meyer's (2003, 2004) * phylogenetic papers on cypraeoideans.
* all these requests were attended in the new manuscript and are better explained below
I am sorry that we cannot be more positive on this occasion, but hope that you appreciate the reasons for this decision.

Yours sincerely,

Geerat J. Vermeij
Academic Editor
PLOS ONE

Additional Editor Comments (if provided):

Reviewers' comments:

Reviewer's Responses to Questions

Comments to the Author

1. Is the manuscript technically sound, and do the data support the conclusions?
The manuscript must describe a technically sound piece of scientific research with data that supports the conclusions. Experiments must have been conducted rigorously, with appropriate controls, replication, and sample sizes. The conclusions must be drawn appropriately based on the data presented.
Reviewer #1: No

---

2. Has the statistical analysis been performed appropriately and rigorously?
Reviewer #1: No

---

3. Have the authors made all data underlying the findings in their manuscript fully available? The PLOS Data policy requires authors to make all data underlying the findings described in their manuscript fully available without restriction, with rare exception (please refer to the Data Availability Statement in the manuscript PDF file). The data should be provided as part of the manuscript or its supporting information, or deposited to a public repository. For example, in addition to summary statistics, the data points behind means, medians and variance measures should be available. If there are restrictions on publicly sharing data—e.g. participant privacy or use of data from a third party—those must be specified.
Reviewer #1: Yes

---

4. Is the manuscript presented in an intelligible fashion and written in standard English?
PLOS ONE does not copyedit accepted manuscripts, so the language in submitted articles must be clear, correct, and unambiguous. Any typographical or grammatical errors should be corrected at revision, so please note any specific errors here.
Reviewer #1: Yes

---

5. Review Comments to the Author
Please use the space provided to explain your answers to the questions above. You may also include additional comments for the author, including concerns about dual publication, research ethics, or publication ethics.

Reviewer #1: The paper has two major components: morphological description of species and cladistic analysis of the morphology. The species descriptions are thorough, but for the new species Macrocypraea rhinoceros, anatomy is apparently based on only three specimens, one male and two female, since only three had the sex stated in the materials examined. This is a small sample size from which to judge range of variation. However, if the stated differences in shell morphology are consistent, that combined with the anatomical data is enough to justify naming a new species. The CT scans are suggestive of differences in shell, but could be accompanied by morphometric data that show clear differences.

In almost a century of expeditions to Trindade only those 3 specimens were collected alive, despite dozens of empty shells, in all kinds of degradation degrees, were collected. It is impracticable to wait for more specimens. For luck, both sexes were represented in those samples and their characters were very uniform, in order to convince us that the anatomical characters clearly represent the species and can show the differences with the remaining ones. In addition, the species is very endemic of a remote oceanic island and is scanty there. Certainly, the species has a limited population which must be protected, and the collect of more specimens should be discouraged.

The cladistic analysis not as well developed as the description of anatomy. The authors do not state what software or methods they used to complete the analysis. The data matrix is largely based on Simone (2004), with addition of scoring for two species: Macrocypraea cervus and Macrocypraea rhinoceros (the putative new species). The single tree resulting from the analysis is supposed to be 286 steps long, but at one point (p. 18, first paragraph of discussion) the manuscript states that length with the same characters as in Simone (2004) , and at another (p. 19) says that three more characters were added to resolve a trichotomy of M. cervinetta, M. zebra and M. rhinoceros that resulted from analyzing the original matrix, but again reports length 286. Also, adding characters specifically to address a particular polychotomy can lead to bias in the analysis. When I reran the analysis in PAUP* 4.0a166 using heuristic search, unordered characters, TBR, 1000 replicates, I got 11 trees of length 280, with little resolution in the Cypraeidae (attached). I did not use the three characters added to resolve the trichotomy. Simone (2004) used PAUP, with ordered characters in some analyses, but did not state which characters were ordered. The matrix from Simone (2004) was supposed to result in a tree of length 281 when Stromboidea was used as the outgroup (p. 174), but I retrieved a length of 279. The analyses in 2004 and in the current manuscript are not reproducible because not enough detail is given about how they were performed.

Attending to the Editor’s suggestion, we removed completely the phylogenetic part of the paper and modified the discussions to a comparative model only, without the phylogenetic inferences.

Even if the analysis were reproducible, it does not include the sister group of Macrocypraea, so the synapomorphies /autapomorphies attributed to Macrocypraea and Lorenzicypraea cannot be confirmed. Meyer (2003, Biological Journal of the Linnean Society 79: 401-459 and 2004, Malacologia 46: 127-156) showed based on molecular data that the sister group of Macrocypraea is Leporicypraea + Mauritia, whereas the current manuscript uses Cypraea and does not treat any member of Leporicypraea or Mauritia. Meyer (2003, 2004) found that Macrocypraea cervus is sister to Macrocypraea zebra on a node with 100% bootstrap support, with M. cervinetta sister to (cervus + zebra). This contradicts the finding in the current manuscript that Macrocypraea zebra groups with M. cervinetta and casts doubt on the need to elevate Lorenzicypraea to full genus status for M. cervus, yet the authors have not cited Meyer's work. Anatomical data from Leporicypraea and Mauritia needs to be added to the cladistic analysis.

Both Meyer's papers (2003, 2004) were consulted and the pertinent data were inserted in the new version. Moreover, the elevation of Lorenzicypraea to the genus rank was also disregarded. Then, all species are maintained in Macrocypraea as the current taxonomy considers.

Another issue is that the authors have not discussed fossils attributed to Macrocypraea, such as M. veintensis Perilliat, Avendano & Vega, 2003, M. spengleri Petuch, 1990, M. joanneae Petuch, 2004 and M. anguillana (Cooke, 1919).

All fossil species of Macrocypraea were searched and their differences were inserted in the new version of the paper (in fifth paragraph of the Discussion). Dr. Edward Petuch, who described most of those species and reviewed the remaining ones, sent to us good photos and papers that helped a lot the analysis.

In the context of a paper entitled "Revisiting the systematics of Macrocypraea", the status of these taxa should be addressed. The authors should also demonstrate that none of the half dozen synonyms of M. zebra are referable to their new taxa, M. rhinoceros.

The context of revision was removed from the present version of the paper. It is now only a detailed comparison on the anatomy and taxonomy of all Macrocypraea species, improving the context of the new species' description. Additionally, we searched all 11 M. zebra synonyms and inserted a discussion on them as the third paragraph of the paper. We realized that no one species was described to the South Atlantic, 3 species were described without locality. Examining all available figures of the original descriptions, all shells were more elongated than the new species, showing being clearly M. zebra instead the new species. This information is now in the paper.

Finally, there is already a cypraeid with that specific epithet: Blasicrura pallidula rhinoceros (Souverbie, 1865). There's no rule against the same name being used elsewhere in the family and there's no risk of them becoming homonyms, but the authors might want to consider a more distinctive name.

To avoid any kind of confusion, the new species epithet was replaced to *M. mammoth*, instead of M. rhinoceros. The etymological explanation is in the end of the new species' description.

---

6. PLOS authors have the option to publish the peer review history of their article (what does this mean?). If published, this will include your full peer review and any attached files.
If you choose "no", your identity will remain anonymous but your review may still be made public.
Do you want your identity to be public for this peer review? For information about this choice, including consent withdrawal, please see our Privacy Policy.
Reviewer #1: Yes: Gary Rosenberg

[NOTE: If reviewer comments were submitted as an attachment file, they will be attached to this email and accessible via the submission site. Please log into your account, locate the manuscript record, and check for the action link "View

Attachments". If this link does not appear, there are no attachment files to be viewed.]
- - - - -

In compliance with data protection regulations, you may request that we remove your personal registration details at any time. (Remove my information/details). Please contact the publication office if you have any questions.

Área de anexos

3

plosone 07:02 (há 2 horas)

Dear Dr. Simone, Thank you for contacting PLOS ONE. We understand from your letter that you are willing to carry out revisions on the manuscript to address the

Luiz Ricardo Lopes de Simone <lrsimone@usp.br> 09:02 (há 14 minutos)

Below the new version of the paper is compared to the previous version in the tracking mode. Every change can be, then, checked.

A new species Revisiting the systematics of *Macrocypraea* (Gastropoda, Cypraeidae)), with description of a new species from Trindade Island, Brazil, including phenotypic differentiation with remaining congeneric species.

Luiz Ricardo L. Simone[1] *

[1]Museu de Zoologia da Universidade de São Paulo. Av. Nazaré 481, 04218-970, São Paulo, Brazil. OrcID: 0000-0002-1397-9823; lrsimone@usp.br

Daniel C. Cavallari[2]

[2]Faculdade de Filosofia, Ciências e Letras de Ribeirão Preto da Universidade de São Paulo

Av. Bandeirantes 3900, 14040-901 Ribeirão Preto, SP, Brazil. dccavallari@gmail.com

*Corresponding author

E-mail: lrsimone@usp.br

ABSTRACT

~~The systematics of~~ *Macrocypraea* *mammoth* is ~~rediscussed based on new phylogenetic data. The morphology of all recent species is redescribed. The descriptions include~~ ~~*Macrocypraea cervus*~~ ~~and~~ a new species~~,~~ ~~*Macrocypraea rhinoceros*,~~ from Trindade, a remote oceanic island located 1160 km off Espírito Santo, Brazil. This isolated species is described in a detailed morphological scenario that includes all Recent congeneric species. The detailed anatomy of two Recent species, *M. zebra* and *M. cervinetta*, were described in a previous paper (Simone, 2004). The remaining one, *M. cervus*, is included herein. Brief taxonomical comments on all these species is also included. The new species~~It~~ can be distinguished from other Western Atlantic species by its larger size, proportionally heavier and more solid shell, more rounded and wider outline, longer posterior tapered ending and slightly inflated base; anatomically it has some exclusivities, such as the mantle papillae mostly bearing 3-5 distal aligned projections, osphradium with a shorter branch, modifications in some odontophore muscles, penis with a clear glandular region, and a sac-like bursa copulatrix with a long duct. Based on the comparative~~The characters of both species studied herein were included in the matrix of a previous paper (Table 1). The resulting topology of the new cladistics~~ analysis~~, reinforces~~ the genus~~monophyly of~~ *Macrocypraea* can be defined~~, supported~~ by ~~four synapomorphies:~~ the wide distance between the osphradium and gill; a~~the~~ twofold buccal muscle (mc); a~~the~~ radular ventral tensor muscle (pair m11) surrounding radular sac; and a bursa copulatrix located at middle level of the pallial oviduct. ~~Based on the resulting topology, we elevate the subgenus~~ ~~*Lorenzicypraea*~~ ~~and the nominal subgenus to genus rank, and~~ ~~*M. cervus*~~ ~~is herein recombined as~~ ~~*Lorenzicypraea cervus*.~~ Register ZooBank: urn:lsid:zoobank.org:pub:C8E6E515-508F-47DE-9753-4EA619C1DDFD

KEYWORDS. Phylogeny, Systematics, Morphology, Anatomy, Western Atlantic.

RUNNING TITLE. Macrocypraea (Mollusca) revisited.

Introduction

The cypraeid genus *Macrocypraea* was introduced by Schilder (1930) in a footnote as a replacement name for *Erythraea* Mörch, 1877, which was preoccupied by Sowerby (1839). It currently encompasses three extant species, namely *Macrocypraea zebra* (Linné, 1758), *M. cervus* (Linné, 1771), and *M. cervinetta* (Kiener, 1844). Recent studies have recognized several subspecies within the group: two subspecies for *M. cervus*, including the nominal subspecies ranging from the Upper Caribbean to the Gulf of Mexico, and *M. c. lindseyi* Petuch, 2013 restricted to southern Cuba; two for *M. zebra*, including the nominal subspecies ranging from Florida to the Caribbean, and *M. z. dissimilis* (Schilder, 1924) distributed along the Brazilian coastline; and two for *M. cervinetta*, from the Pacific, namely *M. c. cervinetta* ranging from Mexico to the western coast of South America, and *M. c. californica* Lorenz, 2017 from the Gulf of California. The genus also currently encompasses two subgenera, including the nominal subgenus, and the monotypic *Lorenzicypraea* Petuch & Drolshagen, 2011 with *M. cervus* as its type species. The genus only occurs in the Tropical and Subtropical coasts of the Americas.

The shell of *Macrocypraea* is mainly characterized by being dorsoventrally compressed with fine, darker-colored apertural teeth, well-developed fossula and columellar sulcus, dark brown dorsum, brown color in general (usually darker around the spire) with dispersed pale spots dorsally and laterally (Simone, 2004; Lorenz, 2017). Anatomically, a set of eight synapomorphies was reported by Simone (2004, 2011) as exclusive of *Macrocypraea* based only on *M. zebra* and *M. cervinetta*, as follows (characters and states from Simone, 2004, in parenthesis): mantle papillae simple, broad, base narrow (30/4); osphradium separated from anterior region of gill (44/1); presence of two bundles of buccal muscles “mc” (67/1); odontophore pair of ventral tensor muscles of the radula m11 with insertion surrounding ventral region of radular sac base (70/2); odontophore pair m7 originating at anterior border of ventral m4 branch (80/1); dorsal inner folds of buccal mass with oblique secondary furrows in their anterior region (94/2); bursa copulatrix U-shaped, bearing inner folds (120/1), located in middle of pallial oviduct (121/1).

Trindade is an oceanic island located ca. 1160 km off the coast of Espírito Santo, southeastern Brazil (20°30'30″S 29°19′30″W). Together with Martim Vaz and three lesser islands, Trindade is an emerged portion of the Vitória-Trindade Chain, a ~6 million-year-old series of extinct underwater volcanoes and guyots spread along the Brazilian shelf. These islands are known to harbor endemic marine mollusk species, e.g., *Leucozonia ponderosa* Vermeij & Snyder, 1998, and *Lottia marcusi* (Righi, 1966), among others (Righi, 1966; Couto & Pimenta, 2012; Salvador, Cunha & Simone, 2013). Since its discovery in the early 16th century, Trindade has received scientific expeditions from many different countries (Almeida, 2002). Material collected in some of these expeditions (e.g., Marion Dufresne MD55) has been described in recent papers and revealed several new mollusk species (e.g., Fernandes et al.,

2013). Four of the latest expeditions carried out by different Brazilian research teams from 2011 to 2015 recovered many live specimens, including three individuals and several shells of a particularly large new species of Macrocypraea, which is introduced herein. Samples of the new species, even shell fragments, are very rare. Finding live specimens is an extraordinary event, which was only made possible after an intense survey during the last expedition.

In a previous phylogeny of the Cypraeoidea, Simone (2004) described the anatomy of *M. zebra* and *M. cervinetta*. The present paper supplements that study with brief taxonomical comments and by thoroughly describing the anatomy of two additional species, including the new species from Trindade Island and *M. cervus*, which was not anatomically described before. These descriptions provide a more accurate scenario for~~. We take~~ the introduction of the new~~opportunity to include the anatomical data revealed herein in the matrix of Simone (2004) in order to obtain an updated morphology-based tree with every extant~~ species, which ~~of *Macrocypraea*. The systematics of the genus~~ is addressed herein, with remarks~~reevaluated based~~ on the genus level. This~~resulting topology. The present~~ paper only deals with fossil *Macrocypraea*~~is, then, a formal revision of the so far considered~~ species for comparative purposes. The ~~of *Macrocypraea*, at least in specific level, as the review in~~ subspecific level is not also addressed here, which would demands a different set of samples and approaches that are out of our~~, away from the present~~ scope.

Material and Methods

Specimens studied herein are adult and subadult individuals recovered by four recent expeditions to Trindade (2011-2015), with the addition of the *Macrocypraea* specimens previously studied by Simone (2004). Additional specimens from older expeditions (1916-1976) and private and museum collections were also examined. Recent specimens are deposited in the Museu de Zoologia da Universidade de São Paulo (MZSP); older items came from the Museu Nacional da Universidade Federal do Rio de Janeiro (MNRJ). Samples of similar species analyzed here are also from the MZSP collection and come from different localities along the Western Atlantic. Abbreviations used for shell measurements: H, height; L, length; W, width.

Specimens used in the CT-Scan analysis were scanned at the Centro para Documentação da Biodiversidade, University of São Paulo, Ribeirão Preto, Brazil, using a GE Phoenix v|tome|x s 240 Industrial High-Resolution CT & X-Ray System. High-resolution x-ray computed tomographies were obtained through a 240 kV high-power microfocus source, using the following settings: no filter, digital high-contrast detector DXR250RT, source operating at

80 Kv and 200 mA, 1 x 1 binning, source to object distance (FOD) 426.149 mm, source to detector distance (FDD) 812.4319 mm, 5 frames averaged, 1 skip frames, 1,300 projections, 333.09 ms timing, default offset correction, default gain correction. Resulting images were 32-bit grayscale and 990 (width) by 1000 pixels (height). Three-dimensional reconstruction was performed with GE Phoenix Datos X2. Visualization, editing, and segmentation of reconstructions of the CT models were carried out in VGStudio Max® V3.0.

~~Data on *Macrocypraea rhinoceros* and *M. cervus* were analyzed and inserted in the data-matrix of Simone (2004: 173). Two lines were added to that matrix (see Table 1) as well as two terminal taxa corresponding to the species described herein. The new matrix was analyzed following the methods of Simone (2004: 27). The modified portion of that cladogram showing *Macrocypraea* and its sister-taxon, *Cypraea tigris* Linné, 1758, is shown in Fig. 57. The remaining cladogram is exactly the same as reported by Simone (2004: figs. 530, 531) and is not illustrated.~~

~~Table 1: Matrix of characters added to the matrix by Simone (2004: 27)~~

~~Taxon\character~~	~~1 2 3 4 5 6~~<br>~~12345 67890 12345 67890 12345 67890 12345 67890 12345 67890 12345 67890 12345 6~~
~~M. zebra~~	~~11210 11010 00111 12100 01011 11014 00101 21211 00111 11121 11111 11012 11000 1~~
~~M. cervinetta~~	~~11210 11010 00111 12100 01011 11014 00101 21211 00111 11121 11111 11012 11000 1~~
~~M. rhinoceros~~	~~11210 11010 00111 12100 01011 11014 20101 21211 00111 11121 11111 11012 11000 1~~
~~L. cervus~~	~~11210 11010 00111 12100 01011 11014 00101 21211 00111 11121 11111 11012 11000 1~~

~~Taxon\character~~	~~6 7 8 9 10 11 12 13~~<br>~~7890 12345 67890 12345 67890 12345 67890 12345 67890 12345 67890 12345 67890 12~~
~~M. zebra~~	~~1112 11111 11211 01111 10111 11110 02111 11111 01101 10000 11101 10000 01110 11~~
~~M. cervinetta~~	~~1112 11111 11211 01111 10111 11110 02111 11111 01101 10000 11101 10000 01110 11~~
~~M. rhinoceros~~	~~1112 11111 11211 01111 10111 11110 02111 11111 01101 10000 11101 10000 01110 11~~
~~L. cervus~~	~~1112 11111 11212 01111 10111 11110 02111 11111 01101 10000 11100 10000 01110 11~~

Abbreviations in the Figures: **aa**, anterior aorta; **ag**, albumen gland; **an**, anus; **ap**, aperture of pallial gonoducts; **ar**, anterior projection of head-foot; **as**, anal siphon; **au**, auricle; **bc**, bursa copulatrix; **ce**, cerebral ganglion; **cg**, capsule gland; **cm**, columellar muscle; **cv**, ctenidial vein; **dd**, duct to digestive gland; **df**, dorsal fold of buccal mass; **dg**, digestive gland; **di**, diaphragmatic septum; **ec**, esophageal gland; **ef**, esophageal folds; **eg**, secondary esophageal gland; **es**, esophagus; **ey**, eye; **ff**, furrowed part of dorsal folds of buccal mass; **fs**, foot sole; **ft**, foot; **gi** gill; **gp**, parietal ganglion; **hg**, hypobranchial gland; **in**, intestine; **ki**, kidney; **km**, membrane between kidney and pallial cavity; **kv**, ventral lobe of kidney; **ll**, left mantle lobe; **m1** to **m14**, extrinsic and intrinsic odontophore muscles; **mb**, mantle border; **mj**, jaws, buccal, and oral tube muscles; **mo**, mouth; **mp**, mantle papilla; **ne**, nephrostome; **ng**, nephridial gland; **nr**, nerve ring; **ns**, nephridial gland vessel inserted at right from nephrostome; **nv**, nerve; **oc**, odontophore cartilage; **od**, odontophore; **os**, osphradium; **ot**, oral tube; **oy**, ovary; **pb**, proboscis; **pc**, pericardium; **pd**, penis groove; **pe**, penis; **pg**, pedal glands anterior furrow; **pl**, penis gland;

pm, pallial muscle (derived from columellar muscle); **pn**, pedal ganglion; **ps**, pallial spermgroove; **ra**, radula; **rh**, rhynchostome; **rl**, right mantle lobe; **rm**, retractor muscle of proboscis; **rn**, radular nucleus; **rr**, anterior triangular sinus of visceral mass connected to pallial floor; **rs**, radular sac; **rt**, rectum; **sa**, salivary aperture; **sc**, subradular cartilage; **sd**, salivary duct; **sg**, salivary gland; **si**, siphon; **st**, stomach; **su**, subesophageal ganglion; **sv**, seminal vesicle; **te** cephalic tentacle; **tg**, integument; **ts**, testis; **vd**, vas deferens; **vi**, visceral ganglion; **vo**, visceral oviduct.

Systematics

Genus *Macrocypraea* Schilder, 1930

Macrocypraea Schilder, 1930: 55. Lorenz, 2071: 286.

Type species: *Cypraea exanthema* Linné, 1767 [= *M. zebra* (L., 1758) see Schilder (1968)] by original designation. Recent, Western Atlantic.

Macrocypraea mammoth~~rhinoceros~~ **sp. nov.**

(Figs 2–21, 27-28, 32-34, 37-46)

urn:lsid:zoobank.org:act:B76C5BC6-595A-4782-A706-6A2BBF1D718F

Cypraea zebra: Rios, 1987: 59 (partim) (non Linnaeus, 1758).

Cypraea (Macrocypraea) zebra: Leal, 1991: 99; Rios, 2009: 123, text fig. (partim) (non Linnaeus, 1758).

Macrocypraea zebra dissimilis: Lorenz, 2017: 289, text fig., pl. 59 (partim) (non Schilder, 1924).

Type specimens: Holotype ♀, MZSP 137544. Paratypes: BRAZIL: off **Espírito Santo**; Trindade Island: Praia dos Cabritos, MNRJ 5059, 1 shell (B. Prazeres col., xii.1975-ii.1976), Calhaus/Farrilhões, MNRJ 5056, 1 shell (J. Becker & A.B. Costa col., 1965, sta. D248/966), Calheta, MZSP 108077, 20°30’37.6”S 29°18’28.1”W, 1 shell (J.B. Mendonça col., 12.vi.2012),

MZSP 137545, 20°30’29.5”S 29°18’37.0”W, 16.3 m, 1 ♂, MZSP 137546, 1 ♂ (shell now broken) (J.B. Mendonça col., 9.vii.2015).

Type locality: BRAZIL, off Espírito Santo state, Trindade Island, Secon/GCIT, 20°30’20.9”S 29°18’43.7”W, 11.6 m (J.B. Mendonça col., 9.vii.2015).

Diagnosis: Shell large, solid and heavy. Posterior ending tapered and long, base slightly inflated. Anterior end flattened, projected, flanking both sides of siphon. Color pattern with large, scattered beige spots, most basal of which presenting dark brown central area. Teeth of inner lip slope long, close from each other, radially disposed, forming nodes preceding canal. Mantle papillae mostly bearing 3-5 small aligned distal projections. Osphradium elongated, with short posterior branch and narrow anterior branch. Odontophore protractor muscles (m1d) composed of single strong dorsal pair of bundles; ventral protractor pair (m10) with multiple branches. Seminal vesicle with uniform width along its length; pallial sperm groove with similar-sized edges. Penis with clear glandular region. Bursa copulatrix bearing long duct, sac-like, with thin-walled terminal region.

Description:

Shell (Figs.2-21, 75-80) large (95–133 mm), inflated, antero-posteriorly elongated, solid and heavy, with tapered anterior and posterior extremities, 1.8 times as long as wide, slightly taller (~10%) than wide (Figs. 2, 8, 12, 16, 76). Surface glossy, color light to dark brown, with lighter-colored longitudinal median dorsal line (usually dislocated to right), and scattered rounded beige spots; spots near base usually larger, some with dark brown (ocellated) central area (Figs. 4, 10, 14). Basal area usually lighter colored, especially around aperture, with dark brown labral teeth (Figs. 3, 10, 11, 17); younger individuals ground color yellowish, with wide orange transverse stripes dorsally (Figs. 20-21). Aperture slit-like, long and narrow, becoming progressively wider toward anterior end (Figs. 3, 9, 11, 75); width ~12–15 % of shell width. Inner lip glossy, bearing 35–42 strong labral teeth, each individual tooth sometimes branching into 2–3 narrower teeth towards interior of aperture; inner slope full of narrow, transverse teeth, radially disposed (virtual focus above middle region of dorsal shell surface; Fig. 79); anterior plate relatively deep concave, tall (~half local height), ventral end of more anterior teeth strongly nodose, with a node at base of ventral-left siphonal edge (Fig. 79). Outer lip bearing 34–41 strong labral teeth. Canal flanked by pair of spatulate long projections (Figs. 6, 77), length ~1/6 of remaining shell length (Fig. 75) relatively horizontal positioned (Figs 79, 80). Shell walls relatively thick (Figs. 76, 80).

Shell variation relatively small. Size normally over 130 mm, but some rare specimens are about 90 mm. pale spots usually small, uniformly splayed (Figs. 2, 8, 12), but sometimes

spots larger (Figs. 16, 18, 19). Ocellated spots usually visible in right side (Figs. 4, 10, 14, 18), rather than left side (Figs. 5, 13), with exceptions (Fig. 19).

Head-foot (Figs. 37, 39): Exposed areas homogeneous dark grayish-brown. Proboscis cylindrical, large, occupying ~70% of haemocoel volume (Fig. 39). Cephalic tentacles long, origin located approximately at ventral lateral surface of snout base (Fig. 37: **te**); proximal half distinctly broader than distal half. Anterior furrow of pedal gland (**pg**) well-developed. Columellar muscle (cm) short (~half whorl long), thin distally. Anterior projection of head solid-muscular (Fig. 37: **ar**), originating in nuchal region, bending toward anterior region, located at left of siphonal canal. Accessory muscle of columellar muscle running along right edge of main muscle, attached to pallial cavity, right margin gradually fading (Fig. 38: **pm**). Haemocoel relatively broad, elliptical. Haemocoel connection with visceral mass (esophageal and aortic passage) located at middle level of left margin of haemocoel (Fig. 39). Two pairs of small retractor muscles of proboscis (Fig. 39: **rm**) originating in ventro-lateral middle region of haemocoel, bordering lateral haemocoel inner surface towards anterior region, inserting along ventral and lateral wall of proboscis; median pair larger than remaining counterparts.

Mantle organs (Fig. 38): Mantle border very large, broad, with left and right lobes (**rl**) exceeding shell aperture, covering shell completely during activity. Outer surface of exposed part of mantle dark grayish-brown in color (Fig. 28), bearing several uniformly distributed papillae (**mp**), except for anterior and posterior regions. Each papilla with base narrower than its middle region, profile broad, rather cylindrical, narrowing distally, with 3–5 transversally-aligned small terminal projections. Siphon small (**si**) distinctly separated from mantle border, shallow, lacking marginal papillae. Anal siphon (**as**) also separated from mantle border, slightly shallower and locaded on opposite side of incurrent siphon. Osphradium (**os**) occupying ~1/15 of pallial cavity roof, relatively large, located at some distance from siphon. Osphradium with 3 branches; anterior branch turned forward toward siphon, length ~70% of osphradium length, narrow, pointed; right branch similar to anterior branch, but slightly broader and shorter; left branch with ~1/3 of anterior branch length, distal end blunt, rounded. Each osphradium branch bipectinate, each filament low and thin, its base almost fully connected to mantle, except for rounded tip bent externally. Osphradium ganglion thick, running along central region of each branch. Distance between osphradium and gill equivalent to osphradium width. Gill (**gi**) occupying ~1/3 of pallial cavity roof, long, strongly curved (concavity located to left), surrounding osphradium. Ctenidial vein relatively broad, width homogeneous along its length; connection with auricle sub-terminal (Fig. 38: **au**) (described below). Each gill filament tall, triangular, tip long, slender and sharp (Fig. 38). Distance between gill and visceral mass narrow in posterior half, successively far from visceral mass and rectum in anterior half. Hypobranchial gland very developed (**hg**), thick, white; occupying area between gill and rectum in anterior 2/3

of pallial roof. Hypobranchial gland supported by transverse, thin septa originating from adjacent mantle. Rectum relatively broad, running along right margin of cavity. Anus siphoned, located close to anal siphon. Genital gonoducts running ventral and at right from rectum, most in floor of pallial cavity (details below).

Visceral mass (Figs. 38, 41): Relatively small, ~3 whorls posterior to pallial cavity. Right anterior region more anterior than left one for ~1/2 whorl, invading pallial cavity. Gonad (**ts**) occupying from second whorl up to pericardial region, surrounding digestive gland on columellar side. Stomach with adjacent esophagus and intestine obliquely occupying central region of first visceral whorl. Digestive gland (**dg**) dark brown, filling space between stomach (and adjacent digestive tubes) and gonad, being more massive anteriorly to stomach in last visceral whorl. Kidney (**ki**) and pericardium as anterior border of digestive gland.

Circulatory and excretory systems (Fig. 41): Heart relatively large, located in anterior-left region of visceral mass (Fig. 38). Auricle elongated, narrow, connecting with ctenidial vein at about ~1/10 before its posterior end, running immersed in mantle, crossing obliquely and dorsally to gill (Fig. 38: **au**), running about equivalent distance inside pericardial cavity, becoming slightly broader toward its insertion in ventricle. Ventricle large (**ve**), walls thick muscular. Aortas running attached to left pericardial wall, anterior aorta (**aa**) ~3 times broader than posterior aorta, running parallel to esophagus. Anterior aorta within haemocoel easily visible up to buccal mass (Fig. 39: **aa**). Kidney narrow, long, located in central and right regions of posterior limit of pallial cavity (Fig. 38). Kidney chamber mostly hollow, intestine running along its dorsal inner surface, digestive gland located in its posterior limit. Kidney lobe (**kv**) single, as solid glandular mass in right-anterior region of kidney, attached to adjacent intestine; gradually becoming flattened mass attached to membrane between kidney and pallial chambers. Nephridial gland triangular (**ng**), occupying ~1/4 of kidney inner space, transversely folded; central vessel well-developed (**ns**), running on right side, ventral to nephrostome. Nephrostome (**ne**) as broad, transverse slit in left region of membrane between kidney and pallial cavity (**km**), close to pericardium.

Digestive system (Figs. 40-42, 44). Proboscis very long, almost as long as haemocoel, occupying ~70% of haemocoel volume (Fig. 39: **pb**). Buccal mass occupying ~1/2 of haemocoel volume. Oral tube relatively long, broad, thickly muscular (**mj**), presenting several muscle layers in different dispositions (Fig. 44: **ot**). Differentiable jaws absent. Pair of dorsal folds of buccal cavity (**df**) very wide, with very narrow furrow separating folds in median line; dorsal folds becoming separated from each other at their middle level, showing series of narrow, longitudinal folds in this region, running in posterior direction longitudinally (Fig. 44: **ff**), flanked by pair of taller folds (**ef**), running up to esophageal gland. Odontophore muscles (Figs.

40, 42): **m1d**) pair of narrow jugal muscles working as dorsal protractor muscles, relatively flattened, originating in anterior-dorsal surface of mouth, close to median line, running in posterior direction, inserting in posterior end of buccal mass; **mc**) buccal sphincter and circular muscles, relatively thin, inconspicuous; **mj**) jaws, oral tube and peri-buccal muscles, portion connected to odontophore working as odontophore protractors, inserting in posterior-ventral region of odontophore cartilages (Fig. 42: mj); **m2**) pair of broad dorsal retractor muscles of buccal mass, originating in ventral-posterior region of inner surface of haemocoel, originating in same of origin of pair of retractor muscle of proboscis (**rm**) (Fig. 12: **m2**), running towards anterior region, attached to lateral surface of esophagus, inserting in lateral-dorsal-posterior surface of buccal mass; **m3**) narrow and very thin pair of muscles, originating in lateral-ventral region of posterior odontophore surface, running dorsally, covering posterior region of odontophore, inserting in dorso-posterior region odontophore close to esophageal origin (Fig. 42: m3); **m4**) large pair of dorsal tensor muscle of radula, originating around origin of mj, broad at origin, running toward dorsum, covering entire inner odontophore structures and cartilages, inserting as two flaps, medial flap smaller (Fig. 42: **m4a**) connecting directly to radular ribbon, lateral flap broader (Fig. 42: **m4**), connecting laterally along subradular cartilage; **m5**) large pair of secondary dorsal tensor muscle of radula (Fig. 42: **m5**), originating in median-posterior region of m4, close to median line, running toward anterior region of odontophore for relatively short distance (1/3 odontophore length), inserting in shorter region of ventral surface of radular sac, just ventrally to insertion of m4; **m6**) horizontal muscle, relatively thin, connecting anterior-ventral margin of both odontophore cartilages for about 1/4 of their length; **m6a**) accessory horizontal muscle, same function and same size as m6, but located behind it at distance equivalent to half its length (Fig. 42: m6, m6a); **m7**) absent; **m10**) pair of ventral protractor muscle of buccal mass, originating in ventral and lateral regions around mouth, very broad and thick at origin, running toward posterior and medial regions of odontophore, becoming narrow and thin at insertion, inserting in postero-ventral level of odontophore (Fig. 40: **m10**); **m11**) narrow pair of ventral tensor muscles of radula (Fig. 42: **m11**) and auxiliary ventral protractor muscle of buccal mass (Figs. 39, 40: **m11**), originating in middle ventral region of inner surface of haemocoel near origin of retractor muscle of proboscis (**rm**), running towards anterior region of odontophore, dorsally to rm, penetrating into ventro-posterior region of odontophore near radular sac (Fig. 40), running further anteriorly, flanking m6 and m6a, inserting in anterior end of radular ribbon; **m12, m13**) absent; **m14**) pair of accessory ventral protractor muscle of buccal mass, slightly thin, same origin as m10 but medial, running in posterior direction, covering m10, gradually becoming narrower, inserting in posterior limit of odontophore on m4 (Fig. 40). Pair of odontophore cartilages long, flattened, somewhat elliptical, most of muscles inserted on their outer surface (Fig. 42: **oc**). Radula long, about 3 times odontophore length (Fig. 39: **rs**), coiled behind buccal mass inside haemocoel. **Radula**

(Fig. 32-34): **rachidian** tooth ~1/6 of width of radular ribbon, central cusp broad, length ~1/2 of rachidian length, blunt, curved inwards, bearing pair of very low secondary cusps opposed to central cusp (Fig. 33); **lateral tooth** as wide as rachidian, flattened, curved inwards and medially, tip acute, carinated at periphery, slightly arched, base flattened, bearing very small secondary cusp at base of lateral edge of main cusp; **inner marginal tooth** similar to lateral tooth, but slightly narrower; **outer marginal tooth** similar to inner marginal tooth, but ~40% narrower and lacking lateral secondary cusp. Salivary glands clustering around anterior and middle esophagus, behind nerve ring (Fig. 44: **sg**), located at left-middle region of haemocoel (Fig. 39). Salivary ducts passing through nerve ring; penetrating in dorsal wall of buccal mass, running immersed along base of dorsal folds; opening at middle level of dorsal folds, near and bent toward median line (Fig. 44: **sa**). Anterior esophagus somewhat narrow, inner surface with pair of tall longitudinal folds (as extension of dorsal folds of buccal mass) (ef), flanking series of longitudinal folds (Fig. 44: **ff**). Middle esophagus broad, smooth; ventral esophageal gland massive, broad, well delimited, located in posterior-right region of haemocoel (Fig. 39: **ec**); inner surface of esophageal gland entirely filled with several thin septa, bearing fragile glandular tissue (Fig. 44: **ec**). Posterior esophagus narrow (Figs. 39, 41: **es**), long (about half of total esophagus length), inner surface lacking folds. Posterior esophagus located inside visceral mass, gradually becoming broader, with smooth inner surface (Fig. 41). Stomach large (Fig. 41), u-shaped, located around penultimate whorl of visceral mass (Fig. 41: **st**); esophagus inserting on its left side with no clear distinction. Duct to digestive gland (Fig. 41: **dd**) located at transition between esophagus and stomach, almost as broad as esophagus, curved anteriorly, bearing series of transverse small folds along its ventral region; septa ending in duct bifurcation, at considerable distance from their origin. Intestine broad at origin, identifiable as right branch of stomach; running in anterior direction and leftward, parallel to posterior esophagus (Fig. 41: **in**); inner surface smooth. Digestive gland described above. Intestine, suddenly becoming bent rightward around left end of kidney, running attached to posterior surface of kidney along its entire length, crossing from left to right side of visceral mass anterior limit (Fig. 41). Rectum short, running along right margin of pallial cavity. Anus broad, siphoned, located close to anal siphon of mantle border (Figs. 38, 41).

Genital system. **Male**. Testis running along columellar surface of visceral mass up to posterior limit of pallial cavity (Fig. 38: **ts**). Seminal vesicle intensely coiled, located in anterior projection of visceral mass, at left of esophagus, as extension of testis (Fig. 38: **sv**). Seminal vesicle with about same caliber along its length. Vas deferens suddenly running perpendicularly in anterior direction along approximately half of visceral mass width (Fig. 38: **vd),** in middle region of right surface of seminal vesicle mass; small aperture to sinus located in region preceding its exit to pallial cavity (Fig. 45: **vd**). Opened sperm grove beginning at its exit to

pallial cavity floor (Fig. 45: **ps**), running up to penis base (Figs. 37, 45), flanked by relatively thick, symmetrical edges. Penis long, with ~1/2 of head-foot length, almost cylindrical, curved distally (Figs. 37, 45: **pe**). Penis groove running along its ventral surface up to penis tip. Penis tip bearing small, broad papilla. Penial gland located in middle region of right edge of penis (Fig. 45: **pl**), disform, occupying ~1/3 of penis surface.

Female (Figs. 43, 46). Ovary occupying approximately same regions than testis of males, but broader posteriorly and very narrow anteriorly (Fig. 43: **oy**). Visceral oviduct (**vo**) running to right, in ventral surface of kidney. Visceral oviduct very slender, short, with ovarian tissue along its length (Fig. 43). In left half of visceral mass oviduct suddenly expanding, becoming thick glandular. Albumen gland of pallial oviduct (**ag**) with pair of glandular laminas, similarly sized, gradually increasing along its length. Bursa copulatrix (**bc**) balloon-shaped, located in middle level of pallial oviduct, insertion right; duct thick walled, as long as local pallial oviduct width, inner surface with 7–8 longitudinal simple folds (Fig. 46: **bc**). Bursa main region wide, with 4–5 spherical lobes, about as wide as local oviduct; walls thin, inner surface smooth (Fig. 46). Bursa orifice located just between both laminas of pallial oviduct. Female genital aperture (**ap**) as simple longitudinal slit, close to floor of pallial cavity far behind anus; edges thick.

Central nervous system. Nerve ring similar to those of remaining species. Degree of fusion of both cerebral ganglia high, almost resulting in single spherical mass. Cerebro-pedal and cerebro-pleural connectives very long, 5–6 times longer than both cerebral ganglia width.

Distribution: Known only from Trindade and Martim Vaz Islands.

Habitat: Rocks and corals, subtidal, ~20 m depth (Figs. 27, 28).

Etymology: The specific epithet is in apposition; it is a reference to mammoths~~rhinos~~, which are widely recognized for their huge size and heavy build, an allusion to the large proportions and heavy shell of new species. Moreover, the~~ and its~~ long anterior projections of the shell are reminiscent of a mammoth's tusk.~~.~~ The epithet also follows the tendency of naming *Macrocypraea*'s after animals.

Measurements (respectively length, height, width in mm): Holotype: 133.1 by 54.6 by 72.4; MZSP 108077: 95.8 by 46.7 by 54.9; MZSP 104754: 116.7; MZSP 108079: 74.1 by 32.6 by 41.1; MZSP 108084: 111.6; MZSP 108095: 114.9 by 56.6 by 65.9. MZSP 101404: 88.7 by 37.1 by 47.4; MZSP 108054: 103.3 by 46.7 by 55.3; MZSP 118452: 111.7 by 49.5 by 60.4. MZSP 121946: 120.1 by 53.1 by 65.0.

Material examined: Types. *Additional material:* BRAZIL. off Espírito Santo; Trindade Island, MNRJ 30770, 30771, 30772, 35142, 4 shells (Bruno Lobo col., 1916), Praia dos Cabritos (Cabritas), MNRJ not numb., 1 shell (A Coelho col., 31.x.1957), MNRJ 5055, 2 shells

(L.S.Silva & J. Becker col., i.1966), MNRJ 5058, 3 shells (J. Becker col., xii.1958), MNRJ 5054, 1 shell (S. Ypiranga, A Coelho & J Becker col., x.1957), MZSP 108079, 1 subadult shell, 20°29’38.2”S 29°19’50.2”W, 10 m (J.B. Mendonça col., 12.vi.2012), MZSP 108054, 1 shell (J.B. Mendonça coll., 17/vi/2012), Praia da Tartaruga, MNRJ 5057, 3 shells (J. Becker col., xii.1958), MZSP 101404, 1 shell+ 1 fragment (D. Abbate & P.O. Lima col., 25.vii.2011), MZSP 121241, 1 shell fragment (Simone et al col., 25.vii.2011), MZSP 104152, 1 subadult shell fragment (C.H. Guimarães col., 27.ii.2012), Calheta, MNRJ 5060, 3 shells (B. Prazeres col., xii.1975-ii.1976), near local lighthouse, 20°29′52.3″S 29°19′15.6″W, 12.9 m, MZSP 118452, 1 shell (J.B. Mendonça coll., 17/iv/2012), 20°30’26.1”S 29°18’44.2”W, MZSP 108095, 1 shell (J.B. Mendonça col.), Praia do Monumento, 20°30’10.3”S 29°20’36.1”W, 15 m, MZSP 121496, 1 shell (J.B. Mendonça col., 2.xi.2014), Ponta Norte, 20°29’18.7”S 29°20’18.3”W, 10 m, MZSP 108078, 1 shell, MZSP 121237, 1 shell fragment (J.B. Mendonça coll., 03/vii/2012), between Calheta and Andradas Beach, 20°30’37.9”S 29°18’32.0”W, 10 m MZSP 104754, 1 fragmented shell, (C.H. Guimarães col., 14.ii.2012); Parcel das Tartarugas, 20°31’01.3”S 29°17’56.9”W, 5 m, MZSP 108084, 1 fragmented shell depth (J.B. Mendonça col., 11.vi.2012), Enseada Noroeste, 20°29’40.9”S 29°20’44.1”W, 12.2 m, MZSP 104744, 2 shell fragments (C.H. Guimarães col., 30.i.2012); Martim Vaz, 20°28’28.42”S 28°51’24.56”W, 21 m, 1 shell (J.B. Mendonça col., 21.vi.2015).

***Macrocypraea cervinetta* (Kiener, 1843)**

Figs. 57-62

Synonymy see Simone (2004). Complement:

Cypraea cervinetta Kiener, 1843: pl. 6, figs 1-2.

Macrocypraea cervinetta: Meyer, 2003: 411; Simone, 2004: 39, figs. 5-6, 71, 144-153; Paulay & Meyer, 2006: 273, tab. 2; Soriano, 2006: 49, tab. 3; Guevara-Fletcher et al., 2011: 8, tab.5; Torreblanca-Ramírez et al., 2012: 286, tab. 1; Landa-Jaime et al., 2013: 1125, tab 1; Torreblanca-Ramírez et al., 2014: 555, tab. 1, fig. 2j(17); Lorenz, 2017: 289, text. fig., pl. 60; Torreblanca-Ramírez et al., 2017: 86, tab. 1, fig. 2(52).

Distribution: Gulf of California, Pacific Central to South America.

Material examined: As in Simone (2004).

Measurements: As in Simone (2004).

Remarks: The only representative of the genus in the Pacific. Conchologically, it can be readily distinguished from the remaining species by the marked anterior widening of the aperture (Figs. 57-58). Most other shell characters such as color, width/height ratio, and general outline overlap with *M. zebra* in varying degrees. The pair of anterior projections flanking canal relatively short (~1/12 of shell length; Figs. 58, 59), left edge oblique. Inner lip slope with relatively short, spaces teeth, mostly positioned perpendicular to longitudinal axis (Fig. 61). Anatomically, it differs from the remaining species of the genus in having a more anterior origin of the auricle; kidney with ventral lobe bearing a mosaic of pores instead of longitudinal folds; narrower rachidian tooth; narrower seminal vesicle; and a straighter penis with a rounder tip (Simone, 2004). Simone (2004) reported no significant anatomical differences between individuals from the Gulf of California (i.e., *M. cervinetta californica*) and the Central-South America (nominal subspecies). Conchological distinctions between the two subspecies were discussed by Lorenz (2017: 290).

Macrocypraea zebra **(Linnaeus, 1771)**

Figs. 63-68

Synonymy see Simone (2004). Complement:

Macrocypraea zebra: Meyer, 2003: 411; Simone, 2004: 35, figs. 1-4, 70, 116-143; Soriano, 2006: 49, tab. 3; Rosenberg et al, 2009: 636; Rios, 2009: 132, text fig. Lorenz, 2017: 288, text fig.

Macrocypraea zebra zebra: Lorenz, 2017, 288, text fig.

Macrocypraea zebra dissimilis: Lorenz, 2017: 289, text fig.

Distribution: North Carolina to southern Brazil.

Habitat: Rocks and corals, subtidal, 0-37 m depth (Rosenberg et al., 2009).

Material Examined: Specimens of *Macrocypraea zebra* listed in Simone (2004), with the addition of the following: DOMINICAN REPUBLIC: Puerto Plata Province; Puerto Plata, 2-3 m depth MZSP 115363, 1 shell (local people coll., XI/2011). HAITI: Nippes Department; Anse-à-Veau Commune, MZSP 115364, 1 shell (local people coll., 1986). BRAZIL: Bahia State; reefs off Alcobaça, 20-25 m depth, MZSP 72653, 20 shells (A. Bordart coll., VII/2004). Rio de

Janeiro State; Arraial do Cabo, 1-2 m, MZSP 73553, 8 shells (P. Gonçalves coll., XII/2003). São Paulo State; Ilhabela, 23°46'S, 45°21'W, MZSP 121109, 20 shells (J. Colella coll., VII/1968). BRAZIL: Santa Catarina State; precise locality unknown, MZSP 38946, 1 shell (De Fiore coll., date unknown).

Measurements (mean values ± standard deviation, in mm; n = 50): H = 26 ± 7, L = 64.5 ± 16.9, W = 33.7 ± 8.7.

Remarks: Based on the specimens analyzed by Simone (2004) and in the present samples, there are no significant anatomical or conchological differences between individuals of the Caribbean (i.e., *M. zebra zebra*) and Brazilian (*M. zebra dissimilis*) populations. As such, there are not enough characters to distinguish these subspecies other than the supposed range gap. Lorenz (2017) mentioned that the division is corroborated by molecular data, bud did not mention any reference or study in progress and provided no additional evidence to endorse the separation. In our understanding, these subspecies must remain invalid until further evidence is presented. The anterior pair of projections franking canal is slightly longer than that of *M. cervinetta*, but shorter than that of *M. mammoth*~~*rhinoceros*~~, about 1/10 of shell length (Figs. 63, 65). The inner lip slope has fewer and shorter teeth than *M. mammoth*~~*rhinoceros*~~ (Fig. 67), disposed perpendicularly to the longitudinal shell axis; those teeth from anterior concave region are interrupted at middle, with weak ventral nodes (Fig. 67). The left edge of canal is more uniform and arched, oblique positioned (Fig. 67, 68).

~~**Genus *Lorenzicypraea* Petuch & Drolshagen, 2011 new stratum**~~

Macro*cypraea* ~~*(Lorenzicypraea)* Petuch & Drolshagen, 2011: 121-122; Lorenz, 2017: 286 (in syn.).~~

~~Type species: *Cypraea cervus* Linné, 1771, OD. Recent, Western Atlantic.~~

~~***Lorenzicypraea***~~ ***cervus* (Linnaeus, 1771)**~~**comb. nov (new stratum).**~~

Figs. 22-26, 29-31, 35-36, 47-56, 69-74

Synonymy see Lorenz (2017). Complement:

Cypraea cervus Linnaeus, 1771: 548.

Macrocypraea cervus: Meyer, 2003: 411; Soriano, 2006: 49, tab. 3; Rosenberg et al., 2009: 636; Lorenz, 2017: 287, text fig., pl. 58.

Macrocypraea (Lorenzicypraea) cervus: Petuch & Drolshagen, 2011: 121-122.

Type locality: not stated.

Diagnosis: Shell large (~120 mm), with very numerous minute whitish spots, never ocellated, aperture widening anteriorly. Mantle lobes papillae small, simple, slender, very numerous. Hypobranchial gland very thick. Osphradium small, with branches very narrow. Kidney with lobe wide, with radial arrangement. Odontophore pair m3 twofold (m3, m3a). Anterior esophagus with distinct gland. Pallial spermoduct with edges strongly asymmetrical. Albumen gland clearly narrower than capsule gland. Bursa copulatrix simple, curved.

Distinctive description:

Shell (Figs. 22-26, 29-31, 69-74). Shell description in Lorenz (2017: 287). Pair of anterior projections flanking canal ~1/8 of shell length (Figs. 69, 71). Inner lip slope with teeth relatively short, perpendicular to longitudinal axis (Fig. 73); anterior concavity preceding canal relatively small (~1/3 of local shell height), lacking ventral nodules (Figs. 72, 74). Left edge of canal oblique (Fig. 73).

Head-foot (Figs. 47, 48). Characters similar to *M. mammothrhinoceros*. Distinctions as follows: Cephalic tentacles more ventrally positioned. Pairs of retractor muscles of proboscis (Fig. 51: rm) of equivalent size. Proboscis much shorter (described below).

Mantle organs (Figs. 49, 50). Similar to *M. mammothrhinoceros*. Distinctions and remarks as follows: Outer surface of exposed part of mantle dark brown, bearing many relatively small, somewhat uniformly distributed papillae (mp). Each papilla mostly cylindrical, distal end tapering up into sharp pointed tip (Fig. 50: mp), base rarely narrower than middle region; profile broad, rather cylindrical. Siphon (si) bearing series of small papillae along its margin. Osphradium (os) much smaller, occupying ~1/30 of pallial roof area, widely separated from siphon and gill. Osphradium with three branches of roughly similar size and shape, each encompassing ~1/2 of total osphradium length. Each gill filament taller, narrower, triangular, tip sharper (Fig. 50: gi). Distance between gill and visceral mass very close to kidney in posterior half, suddenly becoming far from kidney in anterior half. Hypobranchial gland thicker (hg).

Visceral mass (Fig. 47, 49, 54). Same characters as *M. mammothrhinoceros*.

Circulatory and excretory systems (Fig. 49). Similar to *M. mammothrhinoceros*.

Digestive system (Figs. 53-54). Most attributes similar to *M. mammothrhinoceros*. Distinctions and notable features as follows: Proboscis much shorter (Fig. 51: pb), length ~1/3 of haemocoel length, volume ~30% of haemocoel volume. Buccal mass occupying ~1/4 of haemocoel volume, protruding beyond proboscis. Pair of dorsal folds of buccal cavity (df) slightly narrower; dorsal folds becoming narrow, separated from each other in their middle level, space between folds bearing two series of narrow, slightly oblique smaller folds (Fig. 53: ff) delimited by pair of taller folds (ef) as continuation from buccal dorsal folds, running along entire middle esophagus, fading in posterior third of esophageal gland (Fig. 53). Odontophore muscles (Fig. 52) also similar, differing by **m1b**) composed of three pairs of narrow jugal muscles, functioning as dorsal protractor muscles, relatively flattened, originating on latero-dorsal surface of mouth, extending postero-dorsally, inserting into dorso-lateral outer wall of buccal mass; **m4d**) absent; **m3**) similar characters but twofold (Fig. 52: m3 + m3a), with inner pair (m3a) smaller and thinner; **m10**, **m11**, **m14**) pairs of similar features, but much narrower and thinner. Radular teeth (Fig. 35-36) also similar, differences as follows: **rachidian** tooth ~30% narrower, terminal cusp curved forwards (instead of inwards); **lateral tooth** with sharper pointed tip, straight (instead of undulated); **both marginal teeth** also similar, straight, with sharper pointed tip. Salivary glands slightly larger (Fig. 52: sg). Salivary ducts also opening in middle level of dorsal folds, but positioned more laterally, at middle of dorsal fold width (Fig. 52; sa). Dorsal folds flanking pair of sets of longitudinal folds (Fig. 53: ff), ending before anterior esophagus. Anterior esophagus bearing lobes of glandular tissue (Figs. 52, 53: eg); inner surface with a pair of tall longitudinal folds (extension of dorsal folds of buccal mass) (ef). Middle esophagus with pair of folds (ef) coming from anterior esophagus, running along large esophageal gland aperture (ec); this pair of folds fading before posterior region of esophagus. Stomach large (Fig. 49), u-shaped, located about in penultimate whorl of visceral mass (Fig. 49: st); esophagus inserting into its left side with no clear distinction. Mid and hindgut (Fig. 49) similar to preceding species. Anus narrow, siphoned (Fig. 49: an).

Genital system. **Male**. Similar to *M. mammothrhinoceros*, except for: branch of testis at left ot seminal vesicle (Fig. 54: inferior ts); Seminal vesicle (Fig. 54: sv) coiled in T-fashion, with its left coils slightly broader than the right ones. Opened sperm grove (Figs. 48, 55: ps), flanked by relatively thick edges, with left edge narrower and taller than right edge mainly in its posterior region. Penis (Figs. 48, 55: pe) slightly broader, lacking penial gland in its middle region.

Female (Fig. 49). Features similar to *M. mammothrhinoceros*. Notable features as follows: Visceral oviduct slightly more convolute. Albumen gland of pallial oviduct (ag) ~1/3 of capsule gland width, with similar length. Bursa copulatrix (bc) also balloon-shaped, but narrower and

simpler, composed of a single blind-sac structure bent to the right; inner surface bearing 7-8 tall longitudinal folds.

Central nervous system (Fig. 56). Cerebral (ce) and pedal (pn) ganglia very close to their counterparts, commissure indistinct. Pair of cerebral ganglia occupying ~1/40 of haemocoel volume. Pleural ganglia (gp) partially fused to cerebral ganglia, with indistinct limits. Supra and suboesophageal ganglia (su) located dorsally, each connected to adjacent cerebral ganglia, size ~1/4 of cerebral ganglion size, bearing connective between them as wide as their own width. Pair of cerebro-pedal and pleuro-pedal connectives relatively symmetrical, long, their length ~twice cerebral ganglia width. Pair of pedal ganglia (pn) ~70% of cerebral ganglia size, almost spherical.

Distribution: South Florida to north of Cuba.

Habitat: Rocks and corals, subtidal, 0-53 m (Rosenberg et al., 2009).

Material examined: USA: **Florida**; Florida Keys, off Marathon (E. Malone col., 1970), MZSP 121238, 1 shell, MZSP 121239, 1 shell, MZSP121240, 1 shell (ex. Colella collection), SE point of Big Pine Key, ANSP 93276 (A6307), 1♀ (H.A. Pilsbry col., 1907, ex C.B. Moore), Bahia Hondo Key, 24°40’04”N 81°15’55”W, ANSP 216746 (A15658), 1♂ (A.J. Ostheimer col., 2.vi.1958); Indian River, off Wabasso, sand flat north of rest area on causeway state route 512, 27°45.943’N, 80°25.257’W, MZSP 46866, 1 shell (Simone col., 5/VIII/2004); Keys, N of Keys Marine Laboratory, 24°49.78’N 80°48.51’W, MZSP 36106, 1♂, 2♀ (Simone col., 22.vii.2002). CUBA: **Cienfuegos** Province; off Cienfuegos city, 2-4 m, MZSP 115362, 1 shell (under dead corals, local people col., vi.2000, ex Femorale). MEXICO: Gulf of Mexico, MZSP 8149, 1 shell (Sowerby col., 1896). COSTA RICA: Western Coast, Tamarindo Bay, left of Hotel Rocky Point. 17°N, 85°20’W, MZSP 131503, 1 shell (B. Cook coll., 1/ii/1982).

Measurements (height, length, width in mm). MZSP 8149: 64.1 by 136.3 by 80.0; MZSP 46866: 36.5 by 71.4 by 41.0; MZSP 115362: 44.2 by 92.2 by 55.5; MZSP 121238: 47.7 by 98.5 by 57.2; MZSP 121239: 44.2 by 95.5 by 55.4; MZSP 121240: 55.7 by 116.2 by 67.7.

Remarks: *Macrocypraea*~~*Lorenzicypraea*~~ *cervus* also has some anatomical exclusivities that have been explored in the present comparative description. Some are particularly interesting, e.g., the slender, small, and more numerous papillae of the mantle lobes (Figs. 49, 50: mp), the thick hypobranchial gland (Fig. 49: hg) reinforced by wide transverse septa, and the very narrow osphradium branches (Fig. 49: os). The wide renal tissue clearly organized in radial folds (Fig. 54: ki), the two-folded odontophore pairs m3 (Fig. 52: m3, m3a), the clear, bulging glandular region of the anterior esophagus (Figs. 52, 53: eg), the strong asymmetry of pallial spermoduct edges (Fig. 55: ps), mainly in its posterior region, the clear distinction of width between the

albumen and capsule glands in the pallial oviduct (Fig. 49: ag, cg), and the bursa copulatrix comprised of a simple, bent sac (Fig. 49: bc) are also noteworthy exceptionalities.

Discussion

Phylogeny

As explained above, the morphological data of the two species studied herein were added to the matrix by Simone (2004). The lines related to the genus *Macrocypraea* are reproduced herein

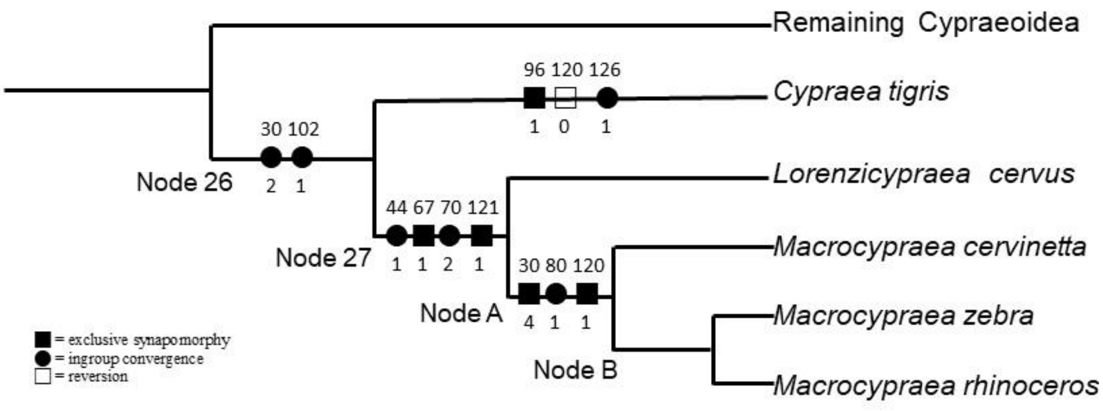

(Table 1). The remaining lines are the same as reported by Simone (2004). The phylogenetic analysis of the updated matrix resulted in a single cladogram, with length = 286, CI = 71, RI = 93. The topology is the same by Simone (2004: figs. 530, 531), differing only by the addition of the two species studied herein at node 27 (Simone, 2004: fig. 531). Since the topology is mostly the same, only the modified portion is illustrated herein (Fig. 81), which includes nodes 26 and 27 (Simone, 2004: fig. 531).

Fig. 81. Portion of the cladogram resulted in the new matrix inserted in that by Simone (2004, fig. 530), with addition of the species studied herein. Only the modified portion of the cladogram is shown. The nodes numeration is the same as in Simone (2004). Synapomorphies of each modified node shown. Length = 286, CI = 71, RI = 93.

Using only the characters listed by Simone (2004), *Lorenzicypraea cervus* resulted as sister species to the species in the *Macrocypraea* branch (Fig. 81: node 27). The remaining three species resulted in a trichotomy (Node A). An additional analysis was performed with the addition of three characters, as follows: A) Anterior shell projection more elongated: exclusive to *M. zebra* and *M. rhinoceros*; B) Ocellated spots at lateral region of the shell: also exclusive to *M. zebra* and *M. rhinoceros*; C) Pallial papillae with base narrower than their middle region, with tip bearing 1-4 small projections: exclusive to *M. zebra* and *M. rhinoceros*. The addition of these 3 characters resulted in a different topology lacking polytomies: *M. zebra* and *M. rhinoceros* ended up in a same branch (Fig. 81: node B) supported by the three above mentioned characters (A-C). In this case, the new cladogram (partly shown in Fig. 81) has length = 286, CI = 71, RI = 93.

Morphology and taxonomy

Individuals of *Macrocypraea mammothrhinoceros* have been reported from Trindade and referred to as *M. zebra* or its older combination *Cypraea zebra* in the literature and museum collections for quite a long time. Even so, most of the specimens deposited in museum collections consist of fragmentary and empty shells. The oldest lots, which are deposited in the Museu Nacional do Rio de Janeiro (MNRJ), date back to 1916. Leal (1991) had already pointed out the large size of this species as distinctive from the continental specimens, regarding it as conspicuous and abundant among beach drift in Trindade. By the time Leal reported his findings, however, no other specimens had been collected in the island since 1976, raising the possibility of extinction. The lack of living specimens and the fact that most of the recovered shells were badly damaged did nothing but reinforce this suspicion. Still, the live individuals collected in the latest samplings nearly a hundred years since the first shells were found at Trindade dismissed this suspicion. They also revealed crucial anatomical information that further distinguished the newly introduced *M. mammothrhinoceros* from its allysister species, *M. zebra*. Some of the samples studied herein with precise collection data were obtained during the daytime, since nocturnal diving in the region is forbidden by the Brazilian maritime authority. It is widely known that related species such as *M. zebra* are mostly active during the night (Crovo, 1971), and we believe the same could be assumed for *M. mammothrhinoceros*. The lack of nocturnal samplings may thus help explain why live specimens were never collected in the past.

Conchologically, *Macrocypraea mammothrhinoceros* is immediately distinguished from the remaining species by its proportionally much heavier shell (Figs. 76, 79, 80), with thick

walls. From *M. zebra* it differs by its wider and more rounded outline (Figs. 75, 79), and color pattern with proportionally larger beige spots. It differs from *M~~L~~. cervus* by having a slightly more elongated outline, with a more tapered posterior end (both laterally and dorso-ventrally, Figs. 77, 79), fewer teeth on the outer lip, and by a color pattern with larger, less coalescent beige spots, the most basal of which can present a dark brown (ocellated) central area. Moreover, *M. mammoth~~rhinoceros~~* differs from *M. cervinetta* in having a much larger and heavier shell, more inflated outline, more pointed anterior projections flanking the siphon area, and more spaced apertural teeth. *M. mammoth~~rhinoceros~~* can be further told apart from both other Western Atlantic cypraeid species by having a longer tapered posterior end and a more inflated base (Figs. 2-21, 75-80). Additionally, the shell of *M. mammoth~~rhinoceros~~* has a longer anterior and more flattened anterior end than those of the remaining congeners; the anterior, siphonal region actually looks like a pair of small spatulas laterally flanking the siphon, projected anteriorly. The closest looking species regarding this character is *M. zebra*, but this pair of anterior projections are shorter and the right one is slightly swollen. Differences in the average number of labial teeth between of the Western Atlantic *Macrocypraea~~/Lorenzicypraea~~* had already been reported, with *M~~L~~. cervus* showing 15% more teeth on the outer lip than *M. zebra* (Crovo, 1971). *M. mammoth~~rhinoceros~~*, is similar to *M. zebra* in this regard, having an equivalent number of columellar and outer lip teeth.

Macrocypraea zebra was so far the single species of the genus that occurred in Brazilian coast. It has 11 nominal synonyms usually found in literature (e.g., Burgess, 1985; Simone, 2004; Rosenberg, 2011), all them described for the North Atlantic and Caribbean. Some synonyms were described without stated locality (*Cypraea dubia* Gmelin, 1791; *C. clauca* Röding, 1789; *C. dissimilis* Schilder, 1924), with no indication of they are based on South Atlantic specimens. Those that were figured show more elongated shells that are comparable to *M. zebra*, rather than *M. mammoth*. Therefore, no synonym of *M. zebra* can be revalidated in order to designate the new species from Trindade.

The CT-Scan examination of the four *Macrocypraea* species (Figs. 57-80) revealed further shell differences. *M. mammoth~~rhinoceros~~* has the thicker shell walls (Figs. 76, 79, 80), which is common in gastropods living in oceanic islands (personal observation), which is possibly an adaptation against the local~~common~~ parrotfishes (Scaridae). The inner slope of the inner lip is particularly informative, as *M. mammoth~~rhinoceros~~* has radially, more densely arranged~~disposed~~, and longer teeth (Fig. 79), while the remaining~~remainder~~ species have the shell region has shorter and more spaced teeth, arranged~~disposed~~ perpendicularly to the longitudinal shell axis (Figs. 61, 67, 73). The density of teeth of *M. mammoth~~rhinoceros~~* is only comparable to *M. cervus* (Fig. 73). The anterior concavity preceding the canal is larger~~largest~~ in *M. mammoth~~rhinoceros~~* (Fig. 79) than the remaining species (Figs. 61, 67, 73, 74), and has

ventral nodes at the end of~~ending~~ the teeth, which are absent in the other species. The highly developed~~higher development of the~~ inner lip slope is visible ~~looking the shell~~ in apertural view (Figs. 3, 9, 11, 17, 75), a region that remains~~remain~~ hidden in the other~~remaining~~ species (Figs. 57, 63, 69). The inner shape~~form~~ of the spire is more spherical in *M. cervus* (Figs. 70, 74), while it is elongated in the remaining species (Figs. 58, 62, 64, 68, 76, 80). The left edge of the anal canal usually is thick in *M. cervinetta* (Fig. 60) and in *M. mammoth~~rhinoceros~~* (Fig. 78), while it is a narrow fold in *M. zebra* (Fig. 66) and in *M~~L~~. cervus* (Fig. 72).

Regarding the fossil species of *Macrocypraea*, *M. mammoth* can be distinguished from *M. veintensis* Perilliat, Avendano & Vega, 2003 (figs. 19-22, Eocene, Mexico), in having a much larger shell (that species is about 40 mm), wider aperture, not so rounded outline, slightly more projected anterior region, and fewer apertural teeth. It differs from *M. spengleri* Petuch, 1990 (figs 5-6; Petuch et al, 2018, figs. 8.2, 8.3) and from *M. joanneae* Petuch, 2004 (Petuch et al, 2018, fig. 8.1) (both Pleistocene, Florida) in lacking an inflated shell, with widely rounded posterior region, narrower anterior region of the aperture, in having fewer apertural teeth, and by the less truncated anterior region; from *M. joanneae*, in particular, *M. mammoth* still differs in having a much more bulged posterior region of the shell, mainly on its left side; and from *M. spengleri*, in particular, in having a narrower aperture, mainly anteriorly. It differs from *M. anguillana* (Cooke, 1919) (Miocene, Anguilla) in being larger (that species is about 30 mm) and having a more visible spire. From *M. trindadensis* (L Miocene, Trinidad) by the larger size and by the slightly wider outline.

Anatomically, *M. mammoth~~rhinoceros~~* can be easily distinguished from the remaining species through the set of characters listed in its diagnosis. Notably, the presence of pallial papillae bearing 3-5 small aligned terminal projections (Fig. 38: mp), as opposed to a single terminal projection in the remaining species. Remarkably, the osphradium in *M. mammoth~~rhinoceros~~* looks somewhat deformed, with a shortened posterior branch as if it were severed, and an elongated and slightly narrower anterior branch (Fig. 38: os). This bizarre osphradium topology was found in all *M. mammoth~~rhinoceros~~* specimens examined herein. The pair of odontophore dorsal protractor muscles (Fig. 40: m1d) also looks exclusive since the remaining species have different arrangements. In *M. cervinetta*, the arrangement is somewhat similar (Simone, 2004, fig. 149: m1d), but much smaller, while in *M. zebra* (Simone, 2004, figs. 123, 125: m1b) and *M. cervus* (Fig. 52), they comprise several small pairs branched far from the median line. The pair of odontophore ventral protractor muscles of *M. mammoth~~rhinoceros~~*, on the other hand, is different in being rather lateralized and bearing 3-4 separated branches (Fig. 40: m10). The folded portion of inner surface of the esophageal origin (Fig. 44: ff) is a single set of folds, running along the anterior esophagus, while this folded region is normally separated into a pair of triangular region on posterior half of the dorsal folds of buccal cavity (Simone,

2004, fig. 124: ff) (Fig. 53: ff). The middle esophagus lacking longitudinal folds flanking the esophageal gland (ec) is also exclusive (Fig. 44: es), which is normally found in the other species (Simone, 2004, fig. 124: es) (Fig. 53). The seminal vesicle of *M. mammoth~~rhinoceros~~* is also very irregularly coiled, as usual for *Macrocypraea*, however, the structure looks with the same width along its length (Fig. 54: sv), while those of the other congeneric species have two clearly uniform regions, a left narrow one, and a wide right one (e.g., Fig. 54: sv). The pallial sperm duct (Fig. 45: ps) is flanked by similar-sized folds, while in the other species an asymmetry is present. The penis of *M. mammoth~~rhinoceros~~* is somewhat as usual for *Macrocypraea* – an elongated, simple structure curved at the tip. However, it has an exclusive well-developed glandular region, bulging in a side of middle penial region (Fig. 45: pl). The bursa copulatrix of *M. mammoth~~rhinoceros~~* is also different in having a long duct and a single wide, thin-walled region at tip (Fig. 46: bc), while in the remaining species the bursa is strongly bent, with a short or absent duct, and bearing strong inner folds (Simone, 2004, figs. 143, 153: bc) (Fig. 49: bc).

With the descriptions of *Macrocypraea mammoth~~rhinoceros~~* and *M~~L~~. cervus*, the anatomy of every extant species of~~previously grouped by~~ *Macrocypraea* has now been described in detail. Even so, the anatomical diagnosis of the taxon remained unchanged. Nevertheless, adding the present data to the morphology-based phylogeny by~~of~~ Simone (2004), which already contained *M. zebra* and *M. cervinetta*, ~~resulted in a tree (Fig. 81) that~~ reinforces the monophyly of *Macrocypraea*.~~/*Lorenzicypraea* (Node 27).~~ The main characters are:~~Node is supported by four synapomorphies (character states as follows):~~ 1) osphradium separated from posterior region of the gill;~~ (44/1);~~ 2) pair of odontophore muscles mc comprising two bundles; ~~(67/1);~~ 3) pair of m11 with insertion surrounding ventral region of radular sac base;~~ (70/2);~~ 4) bursa copulatrix located at middle level of pallial oviduct. Another set of character are present in remaining tree species excepting *M. cervus*, as follows:~~In the topology presented herein, the first branch of *Macrocypraea* is *M. cervus*, separated from the other three species by three synapomorphies (Node A):~~ 1) pallial papillae stubby, broad, with narrow base;~~ (30/4);~~ 2) odontophore pair m7 originating at anterior border of ventral m4 branch;~~ (80/1);~~ 3) bursa copulatrix U-shaped, bearing inner folds. A close relationship of *Macrocypraea* ~~(120/1). With the inclusion of the three extra characters discussed in the Phylogeny section above, *M. zebra* and *M. rhinoceros* resulted in a proper node (Fig. 81, Node B). Otherwise, both species resulted in a trichotomy~~ with ~~*M. cervinetta*. Moreover,~~ *Cypraea* s.s. was obtained by morphological phylogenetic approach (Simone, 2004), and it was somewhat corroborated by molecular approach (Meyer, 2003, fig. 4; 2004, fig. 2), although other ~~*tigris*, the type species of the genus, resulted as the sister taxon to *Macrocypraea/Lorenzicypraea* node, a branch supported by~~ two genera not~~synapomorphies: **1)** pallial papillae simple and long (30/2); **2)** presence of several~~

transverse septa in the anterior duct of the digestive gland (102/1). The emergence of *L. cervus* as the first branch of previously considered *Macrocypraea* is compatible with the validity of the subgenus *Lorenzicypraea* by Petuch & Drolshagen (2011), elevated to generic rank herein, with the remaining three species included in the morphological study resulted still closer – *Leporicypraea* Iredale, 1930, and *Mauritia* Troschel, 1863nominal genus, as contains the type species *M. zebra*.

The discovery of a new cypraeid species is an uncommon event in recent times and serves two immediate purposes. First, it is as a reminder of how poorly known the Brazilian mollusk fauna is, particularly in remote areas such as oceanic islands. Second and most importantly, it draws attention to the necessity of protecting these animals, which, because of the endemicity and rarity, may become endangered in the near future.

Acknowledgments

We are grateful to Marcos Tavares (MZSP), coordinator of the ProTrindade Project (CNPq 403940/2012-5 and FAPESP 2016/50373-6), who made the specimens of *M. mamooth* available for study andthankful to Joel Braga de Mendonça Jr. for conducting the field work at the Trindade Island andJunior, who collected the live individuals. This is the contribution number 8 of the ProTrindade Project. The authors gratefully acknowledge support by the Centro para Documentação da Biodiversidade (Center for Biodiversity Documentation), Department of Biology, FFCLRP, Brazil. This article constitutes part of the project 'Core-facility for the conservation of scientific documentation: biological collections and high technology research in comparative morphology' (CT-INFRA 01/2013), financed by the Funding Authority for Studies and Projects (FINEP), Ministry of Science, Technology, Innovations and Communication, Brazilian Federal Government.

References

Almeida, FFM. Ilha de Trindade - Registro de vulcanismo cenozóico no Atlântico Sul. In: Schobbenhaus C, Campos DA, Queiroz ET, Winge M, Berbert-Born MLC (Eds). Sítios geológicos e paleontológicos do brasil. Vol. 1. Brasília (Brazil): Comissão Brasileira de Sítios Geológicos e Paleobiológicos (SIGEP), DNPM/CPRM, 2002, pp. 369-377.

Burgess, CM. Cowries of the world. Gordon Verhoef publ. Cape. 1985; 289 pp.

Couto DR, Pimenta AD. Comparative morphology of *Leucozonia* from Brazil (Neogastropoda: Buccinoidea: Fasciollariidae). Amer Malacol Bull. 2012; 30(1): 103-116.

Crovo ME. *Cypraea cervus* and *Cypraea zebra* in Florida – One species or two? Veliger. 1971; 13(3): 292-295.

Fernandes MR, Pimenta AD, Leal JH. Taxonomic review of Triphorinae (Gastropoda: Triphoridae) from the Vitória-Trindade Seamount Chain, southeastern Brazil. Nautilus. 2013. 127(1): 1-18.

Guevara-Fletcher CE, Kintz JRC, Mejía-Ladino LM, Cortés FA. Benthic macrofauna associated with submerged bottoms of a tectonic estuary in Tropical Eastern Pacific. J Marine Biol. 2011; 1–13. https://doi.org/10.1155/2011/193759

Landa-Jaime V, Michel-Morfin E, Arciniega-Flores J, Castillo-Vargasmachuca S, Saucedo-Lozano M. Moluscos asociados al arrecife coralino de Tenacatita, Jalisco, en el Pacífico central mexicano. Revta Mexic Biodiversidad. 2013; 84: 1121-1136.

Leal JH. Marine prosobranch gastropods from oceanic islands off Brazil: species composition and biogeography. Oegstgeest: Universal Book Services; 1991.

Linnaeus C. Vermes; pp 543-552. Mantissa Plantarum. Holmiae; 588 pp. 1771.

Lorenz F. Cowries – A Guide to the Gastropod Family Cypraeidae. Vol. 1 – Biology and Systematics. Harxheim: ConchBooks, 644 pp; 2017.

Meyer CP. Molecular systematics of cowries (Gastropoda: Cypraeidae) and diversification patterns in the tropics. Biol J Linn Soc. 2003; 79: 401-459.

Meyer CP. Toward comprehensiveness: increased molecular sampling within Cypraeidae and its phylogenetic implications. Malacologia. 2004; 46(1): 127-156.

Paulay G, Meyer C. Dispersal and divergence across the greatest ocean region: Do larvae matter? *Integrat Compar Biol.* 2006; 46(3): 269-281. https://doi.org/10.1093/icb/icj027

Perrilliat MdC, Avendaño J, Vega FJ. Middle Eocene Cypraeoideans from the San Juan Formation, Chiapas, southern Mexico. Revta Mexic Cienc Geol. 2003; 20(1):41-51.

Petuch EJ. New gastropods from the Bermont Formation (middle Pleitocene) of the Everglates Basin. Nautilus. 1990; 104(3): 96-104.

Petuch EJ, Drolshagen M. Compendium of Florida fossil shells, vol 1. MdM Publishing. Wellington, Florida, 412 pp; 2011.

Petuch EJ, Berschauer DP, Myers RF. Jewels of the Everglades. The fossil cowries of southern California. San Diego Shell Club. 2018; 248 pp.

Petuch, EJ & Drolshagen, M. 2011. Compendium of Florida fossil shells. MdM Publ. Liuzhou, 1: 412 pp.

Righi G. On the Brazilian species in the *Acmaea subrugosa* complex (Gastropoda: Prosobranchia: Patellacea). Malacologia. 1966; 4(2): 269-295.

Rios EC. Compendium of Brazilian Sea Shells. Rio Grande, Evangraf; 2009.

Rios EC, Calvo IS, Barcellos LJ. Moluscos marinos de Isla Trinidad. Comunic Soc Malaco Uru 1987; 7(52-53): 57-62.

Rosenberg G. Malacolog Version 4.1.1. 2011. Website http://www.malacolog.org/index.html (extracted 28.x.2019).

Rosenberg G, Moretzsohn F, García EF. Gastropoda (Mollusca) of the Gulf of Mexico, Pp. 579–699. In: Felder DL, Camp DK (eds.). Gulf of Mexico–Origins, Waters, and Biota. Texas A&M Press, College Station, Texas; 2009.

Salvador RB, Cunha CM, Simone LRL.. Taxonomic revision of the orthalicid land snails (Pulmonata: Stylommatophora) from Trindade Island, Brazil. J Nat Hist 2013; 47(13-14): 949-961.

Schilder FA. Remarks on type specimens of some recent Cypraeidae. J Moll Stud 1930; 19(1): 49-58.

Schilder FA. Linnaeus' type specimens of cowries. Veliger. 1966; 9(2): 91-100.

Schilder FA. The generic classification of cowries. Veliger. 1968; 10(3): 264-273.

Simone LRL. Morphology and phylogeny of the Cypraeoidea (Mollusca, Caenogastropoda). Papel Virtual. Fapesp. São Paulo, 185 pp; 2004 [www.moluscos.org/trabalhos/2004/Simone%202004%20-%20Cypraeoidea.pdf].

Simone LRL. Phylogeny of the Caenogastropoda (Mollusca), based on comparative morphology. Arquiv Zool. 2011; 42(4): 161-323. [www.moluscos.org/trabalhos/Caenogastro/Simone%202011a%20Caenogastropoda%20Phylogeny%20LIGHT.pdf]

Soriano JL. Consideraciones taxonómicas sobre la familia Cypraeidae Rafinesque, 1815 (Mollusca: Caenogastropoda), con una nueva propuesta de clasificación supragenérica. Spira. 2006; 2(1): 41–62.

Torreblanca-Ramírez C, Flores-Garza R, Flores-Rodríguez P, García-Ibáñez S, Galeana-Rebolledo L. () Riqueza, composición y diversidad de la comunidad de moluscos asociada al sustrato rocoso intermareal de playa Parque de la Reina, Acapulco, México. Revta Biol Mar Oceanog. 2012; 47(2): 283-294.

Torreblanca-Ramírez C, Flores-Garza R, Flores-Rodríguez P, García-Ibáñez S, Michel-Morfin JE, Rosas-Acevedo JL. Gastropods with economic potential associated with the rocky intertidal zone of the Marine Region Priority 32, Mexico. Revta Biol Mar Oceanog. 2014; 49(3): 547-557. https://dx.doi.org/10.4067/S0718-19572014000300011

Torreblanca-Ramírez C, Flores-Garza R, Flores-Rodríguez P, García-Ibáñez S, Michel-Morfin JE, Rosas-Acevedo JL, Valdés-González A. () Composition of Prosobranchia-Pulmonata (Mollusca: Gastropoda) in rocky intertidal zone in the Marine Priority Region 32, Guerrero, Mexico. Revta Biol Mar Oceanog. 2017; 52(1): 81-93. https://dx.doi.org/10.4067/S0718-19572017000100006.

Legends

Fig. 1. Schematic map of Americas showing approximate extension of the four studied species, color as indicated.

Figs 2-15. *Macrocypraea* mammoth~~rhinoceros~~ shells of types. 2-7) Holotype MZSP 137544 (L 133.1 mm). 2) Dorsal view. 3) Apertural view. 4) Right view. 5) Left view. 6) Anterior view. 7) Posterior view. 8-10) paratype MZSP 137545 (L 100.0 mm). 8) Dorsal view. 9) Apertural view. 10) right view. 11-15) Paratype MZSP 137546 (now broken) (L 112.3 mm). 11) Apertural view. 12) Dorsal view. 13) Left view. 14) Right view. 15) Same, right portion of shell removed.

Figs 16-31. *Macrocypraea* spp shells and in situ. 16-19) *M. mammoth~~rhinoceros~~* paratype MZSP 108077 (L 95.8 mm). 16) Dorsal view. 17) Apertural view. 18) Right view. 19) Left view. 20) *M. mammoth~~rhinoceros~~* MZSP 108079 subadult specimen (L 80.4 mm), dorsal view. 21) Same, apertural view. 22) *M. cervus* MZSP 8149, from Mexico (L 152.9 mm), Apertural view. 23) Same, dorsal view. 24-26) *M. cervus* ANSP 93276, from Florida (L 129.4 mm). 24) Apertural view. 25) Dorsal view (broken). 26) Same, specimen and dorsal shell wall removed. 27) Alive paratypes in situ.; 28) Alive holotype in situ. 29-31) *M. cervus*, MZSP 115362, from Cuba (L 98.5 mm). 29) Apertural view. 30) Dorsal view. 31) Right view.

Figs 32-36. *Macrocypraea* spp radulae in SEM. 32) *M. mammoth~~rhinoceros~~* paratype MZSP 137546; 33-34) *M. mammoth~~rhinoceros~~* holotype. 35) *M. cervus* ANSP 93276. 36) Same, detail. scales= 32-35: 1 mm, 36: 0.5 mm.

Figs 37-38. *Macrocypraea* mammoth~~rhinoceros~~ anatomy. 37) Head-foot, male, right view. 38) Pallial roof and visceral mass, ventral view, portion of gill ventral to pericardium removed. Scales = 10 mm.

Figs 39-42. *Macrocypraea* mammoth~~rhinoceros~~ anatomy. 39) Head, ventral view, foot and columellar muscle removed, radular sac uncoiled downwards. 40) Buccal mass, left view. 41) Visceral mass and posterior part of pallial cavity, ventral view, midgut seen as in situ, kidney opened longitudinally, only topology of gill shown. 42) Odontophore, dorsal view, both cartilages (oc) deflected, left m4 and m5 (right in Fig.) partially sectioned. Scales = 5 mm.

Figs 43-46. *Macrocypraea mammothrhinoceros* anatomy. 43) Female genital system and adjacent structures as in situ, ventral view, transverse section artificially done in middle region of pallial oviduct, ovary (oy) only partially shown, kidney not shown in details. 44) Foregut, ventral view, opened longitudinally, odontophore (od) deflected to left. 45) Anterior male genital structures, dorsal view, topology of right tentacle (te) also shown. 46) Detail of middle region of pallial oviduct, dorsal view, bursa copulatrix (bc) artificially sectioned in two regions. Scales = 5 mm.

Figs 47-51. *Lorenzicypraea cervus* anatomy. 47) Whole specimen extracted from shell, left view, visceral mass slightly deflected upwards. 48) Head-foot, male, right view.; 49) Pallial roof and visceral mass, ventral view, portion of gill ventral to pericardium removed, anterior region of digestive gland (dg) and hypobranchial gland (HG) partially sectioned transversally, transverse section of indicated region of capsule gland (cg) also shown. 50) Right mantle lobe, middle region, outer view. 51) Head, ventral view, foot and columellar muscle removed. Scales = 5 mm.

Figs 52-53. *Lorenzicypraea cervus* anatomy. 52) Foregut, left view, some structures deflected, 53) Same, ventral view, opened longitudinally, odontophore removed. Scales = 5 mm.

Figs 54-56. *Lorenzicypraea cervus* anatomy. 54) Visceral mass, male, ventral view, partially uncoiled. 55) Right region of pallial cavity floor and penis, dorsal-slightly posterior view, transverse section in two indicated regions also shown. 56) Central nervous system, ventral view. Scales = 5 mm.

Figs 57-68. *Macrocypraea*, 3D reconstructions of shells (CT-Scan). 57-62) *M. cervinetta* MZSP 131503 (L 58 mm). 57) Frontal view. 58) Same, more ventral portions removed. 59) Anterior view; 60) Posterior view. 61) Right view, right half removed. 62) Same, longitudinal section along columellar axis. 63-68) *M. zebra* MZSP 38946 (L 100 mm). 63) Frontal view. 64) Same, more ventral portions removed. 65) Anterior view. 66) Posterior view. 67), Right view, right half removed. 68) Same, longitudinal section along columellar axis.

Figs 69-80. *Macrocypraea* and *Lorenzicypraea*, 3D reconstructions of shells (CT-Scan). 69-74) *ML. cervus* MZSP 121238 (L 98.5 mm). 69) Frontal view. 70) Same, more ventral portions removed. 71) Anterior view. 72) Posterior view. 73), Right view, right half removed. 74) Same, longitudinal section along columellar axis. 75-80) *M. mammothrhinoceros* MZSP 137545 (L 100.0 mm). 75) Frontal view. 76) Same, more ventral portions removed. 77) Anterior view. 78) Posterior view. 79) Right view, right half removed. 80) Same, section along columellar axis.

~~**Fig. 81. Partial cladogram** generated from the new matrix combining the present data and data from Simone (2004, fig. 530). Only the modified portion of the topology is shown. Numeration of nodes follows Simone (2004). Synapomorphies of each modified node are also shown. Length = 283, CI = 68, RI = 89.~~
