# Supplementary material for: A new species of Macrocypraea (Gastropoda, Cypraeidae) from Trindade Island, Brazil, including phenotypic differentiation from remaining congeneric species
Source: PLoS One. 2020 Jan 8;15(1):e0225963. doi: 10.1371/journal.pone.0225963 (PMC6948752; doi:10.1371/journal.pone.0225963)
Supplement: Peer review history

# Author comment: Author response to previous submission

**DOI:** 10.1371/journal.pone.0225963.r001

8 Nov 2019

Attachment Submitted filename: Rebuttal letter Macrocypraea2.pdf Click here for additional data file.

# Editor report: Decision Letter 0

**DOI:** 10.1371/journal.pone.0225963.r002

**Authors:** Geerat J. Vermeij

18 Nov 2019

A new species of Macrocypraea (Gastropoda, Cypraeidae) from Trindade Island, Brazil, including phenotypic differentiation with remaining congeneric species.

PONE-D-19-30595

Dear Dr. Simone,

We are pleased to inform you that your manuscript has been judged scientifically suitable for publication and will be formally accepted for publication once it complies with all outstanding technical requirements.

Within one week, you will receive an e-mail containing information on the amendments required prior to publication. When all required modifications have been addressed, you will receive a formal acceptance letter and your manuscript will proceed to our production department and be scheduled for publication.

Shortly after the formal acceptance letter is sent, an invoice for payment will follow. To ensure an efficient production and billing process, please log into Editorial Manager at https://www.editorialmanager.com/pone/, click the "Update My Information" link at the top of the page, and update your user information. If you have any billing related questions, please contact our Author Billing department directly at authorbilling@plos.org.

If your institution or institutions have a press office, please notify them about your upcoming paper to enable them to help maximize its impact. If they will be preparing press materials for this manuscript, you must inform our press team as soon as possible and no later than 48 hours after receiving the formal acceptance. Your manuscript will remain under strict press embargo until 2 pm Eastern Time on the date of publication. For more information, please contact onepress@plos.org.

With kind regards,

Geerat J. Vermeij

Academic Editor

PLOS ONE

Additional Editor Comments (optional):

I am accepting this very nice paper pending two minor corrections. In the title, please substitute the word "with" by "from". In the discussion, I think you meant to write M. trinidadensis rather than M. trindadensis; please give the author of this species.

Reviewers' comments:

# Editor report: Acceptance letter

**DOI:** 10.1371/journal.pone.0225963.r003

**Authors:** Geerat J. Vermeij

18 Dec 2019

PONE-D-19-30595 

A new species of *Macrocypraea* (Gastropoda, Cypraeidae) from Trindade Island, Brazil, including phenotypic differentiation from remaining congeneric species. 

Dear Dr. Simone:

I am pleased to inform you that your manuscript has been deemed suitable for publication in PLOS ONE. Congratulations! Your manuscript is now with our production department. 

If your institution or institutions have a press office, please notify them about your upcoming paper at this point, to enable them to help maximize its impact. If they will be preparing press materials for this manuscript, please inform our press team within the next 48 hours. Your manuscript will remain under strict press embargo until 2 pm Eastern Time on the date of publication. For more information please contact onepress@plos.org.

For any other questions or concerns, please email plosone@plos.org. 

Thank you for submitting your work to PLOS ONE.

With kind regards,

PLOS ONE Editorial Office Staff

on behalf of

Dr. Geerat J. Vermeij 

Academic Editor

PLOS ONE
